# Supplementary material for: Targeting Yezo Virus Structural Proteins for Multi-Epitope Vaccine Design Using Immunoinformatics Approach
Source: Viruses. 2024 Sep 3;16(9):1408. doi: 10.3390/v16091408 (PMC11437474; doi:10.3390/v16091408)
Supplement: Supplementary file 1 [file viruses-16-01408-s001.zip › Supp. figure S1.pdf]

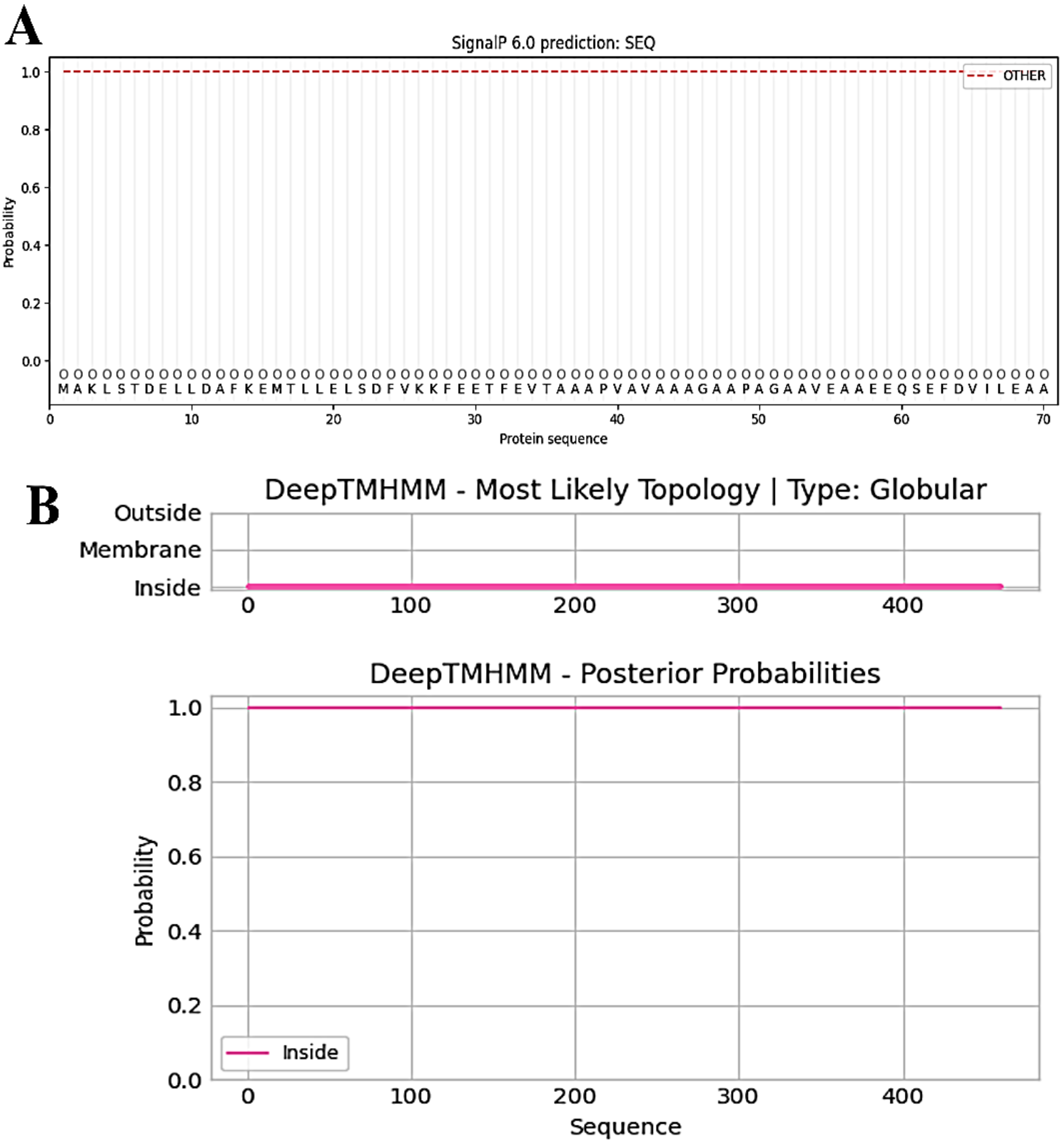

Supplementary figure S1. (A) The plot about the signal peptide prediction of the vaccine. (B) The plot depicts the absence of the transmembrane helix of the vaccine.
