# Supplementary material for: Targeting Yezo Virus Structural Proteins for Multi-Epitope Vaccine Design Using Immunoinformatics Approach
Source: Viruses. 2024 Sep 3;16(9):1408. doi: 10.3390/v16091408 (PMC11437474; doi:10.3390/v16091408)
Supplement: Supplementary file 1 [file viruses-16-01408-s001.zip › Supp. figure S2.pdf]

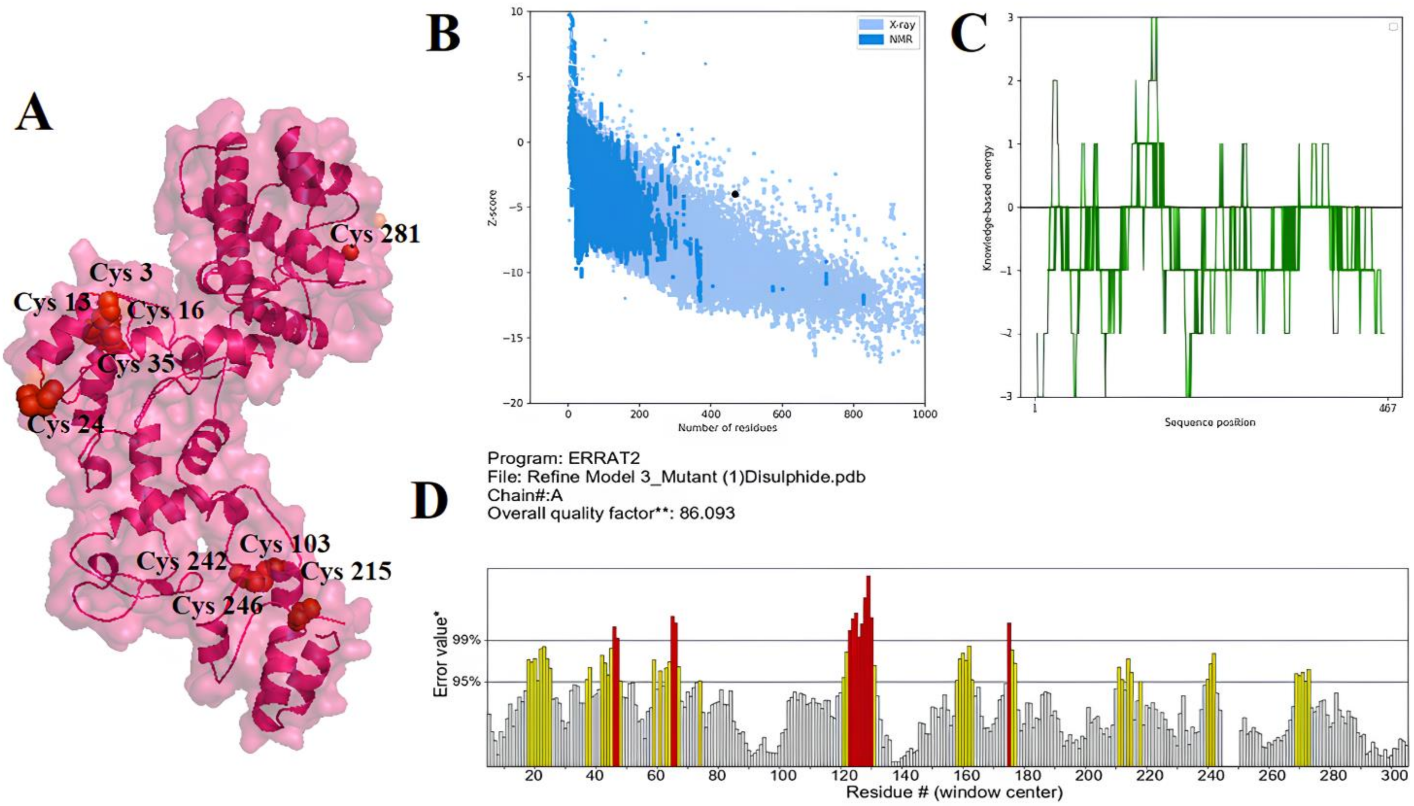

Supplementary figure S2. (A) Illustrates the anticipated 3D structure of the model vaccine construct with a disulfide bond. (B, C) shows the ProsaWeb Z-score. (D) Shows the ERRAT score.
