# Supplementary material for: Targeting Yezo Virus Structural Proteins for Multi-Epitope Vaccine Design Using Immunoinformatics Approach
Source: Viruses. 2024 Sep 3;16(9):1408. doi: 10.3390/v16091408 (PMC11437474; doi:10.3390/v16091408)
Supplement: Supplementary file 1 [file viruses-16-01408-s001.zip › Supp. Table S1.pdf]

**Supplementary Table S1:**
>WPF56447.1 RNA-dependent RNA polymerase, partial [Yezo virus]
MIELLTGEGVIPGYRVSQKLYEDSRSIKKDLRVTPNLQLNIAGRLENYLNSTDARVSDWSEVQQILDESGCSNIDVQLE<br>QKTLEDRVSWRCLLSKISNRAPSMLTLRTLVSLVGSETIPNFLSGLLVH
>YP_010840881.1 nucleoprotein [Yezo virus]
MARLIEAKTREELDKWIHNLDTTHFLGLNTKYTKSLALQPCEPGSCPEAYISFDGKKSDELDAILSGALVDSLKFAAPI<br>PECAWNLTNKIFTEGMAWFNANKDQKCMAWDKKYDTLRSTLPSPEDVAQYQIAARKWRKDIGYECSPNNGIMRGE<br>VTKVYIAPKKYVNDMILMVTDMRSKRRHRLGITEEQEQEALSRKGAEHAPWVEDWVKGGEDRIFSLPSWGGWNK<br>KTKKGLLLGGTAVAHLVQKKVITLKIFRSKVEKAGELLNNQQKMDELGIDVSQARLMHAHMEGCLKEAEGLIKDSV<br>TSAYVQQGSALDTAFSTYYWMWKADVTLANFGALNEMAFLYGQKPVGQKKLLDTLKGTAYKWGSTLANMCATG<br>DFNGERVHMHPGVFTPHRMSEMTAAIGAFPLSNPVKIKEGSASYRYLTNLKTSESNPAAKVVCELFNTHRRAHSDWN<br>SPTSIVPPEHYFHQSLLERLGPFCHVSLVRGNALRVNIVEEMGTGGNP
>YP_010840880.1 RNA-dependent RNA-polymerase [Yezo virus]
MAGKLSKRPELTWEHRINNLFVASIVMPVDESFKVVRMPGDGKCFFYSVSHNMFGTIQGHTRVKWMVRDFAEKHW<br>GLLTEAKEFYRSREEYIRDLNRETYWGGSIEAEILSLRTNQPIIIWRAEDGYTITSASMWRGDSGKGEINLIQSHNNHFD<br>AAYPPELAGTTSGPAVSREDKTVLEAVGETIIDEDDESDTEMEVAKLEDTFLPTYESERIELLSKPTMPPVGPVRRLPAR<br>LEEMLVNEQTLPGKVGRHLNKLYSCNVSVLFINPGEALLIPSHSEVGGKVSISELGRLWLNPSKRIRKENLRTVIAISDQ<br>LMAYVDKGYVLRTFVPKTFISRHAGLVPPDLVRDMTLTATVILLSTFLYKSRMVEKRTFIRACLRDEGRISGKKVSRIL<br>NKLTPHTLYNDPLSVVRQICLAMFGEECGRLVDLFSCLGPVSYLSLSSLELDSLTRNKYYQVLEELSRADTEDFDFSSK<br>EISEIKATVDCCKALREASDKESCEQATGTVLQILGISRQELPRRAKDNRREQEKLVINKFFNDRAISKFVSLQGRAYSG<br>TNLSNILSYTHNLLMSKDKFKLTEDDVGQLELEQRKIRAMLAKEERVPIAIICSMMENRFNNLFKRLPPSCAQECEVLF<br>DSIRNAESYASAWRSALRLKGVAYEGMFALKYNCTYIPEDQKPTLSMIVQTYYPDKFVDFLDKTQKRPEIRDYTPDFS<br>ICQMIVEHAEGLVPPKVSSVQLQAHDLEESGVKLRRTKEKAFPLPEVPIKELRSLDRLKELLLGTGKQNQPVHLKTEK<br>YDESDCTVTSREIIAIEVGYQTDVDGKVFSDTHKWQEAIKLLSKLGIKALVHICADSSSTRLSDWWISPEDARLLKESI<br>SFLFHEMGENSPSDVTDIMVANISTTKVRAQLKSGSIVKTPVTTKDVAAAWNAKLEKIRVRPTGAVLTDGLMDTVKK<br>ALVEGVTMDRASCEKVIEEVVIKNMDEIISLVEETKNKHEVTRNYVTANRLILGWLMEDIECCRCKACVAMIKDAIK<br>GIASEQESVKYLCSQIQLSEHLPCCHTDLPSVKPRSLFSTRCPDITTVRHMTGDGSTDDSELTELDKAIRLTLPGKTERE<br>RKIKRGLEQLIRLSMSDSHIPCIKLPTGQILINRHAFNPTKVSEKRAAKNDEFRDSKLERLTKLLSKERLTGYSEKACKI<br>MNTLLEKTHLQGNSRCCLKPEWAQSVLMDLKTDRNEIDIATKLKETSEKRLNFIVNNDKIIPVSNSEIRQFIVDKSDTIL<br>NRKKNSKLFNLDCLIFKETVLEVLRRLNGTPYFDCIEIIATLTKLLLSFDWFQELVLYSKLCETFLQCCTEFNRSGIKVR<br>RVRHTSINLAIQLPSNKKENMKCCCYNLDFSRNKSTFLMNRRVAVLGAAYPYILIITTLQCLQHQRCLDELTKEHASLQ<br>RDILTANAKLLDLLQSTLCKANLGSFEQAALELRRHCKQTGNFLDHSSYDMIVNTLSGLSVVFGLALGDTLLLNSQPF<br>NKQIQNMRFTMLSGLSRLVSPLELGKKLSSSSRRIESGVSRLYLQLISYCCVEQVEQNSENWKQFDLCPNVFMPSLSIF<br>GMNTSGDRQLIFDIYLVHIYNKEMDNFDEGCIKVLEETAEKHMSWELSLMNDLEKKTKKHERHARLLLGLPTLPRHE<br>LPTEDAIPEDSFSTSSSTSAISSTRSFSSGKNIKTLFGRLKSEIKPFVVEDCLEVSRDELNDFKQVISDVGRGYSYEPNLQ<br>RLYKNVKTIIKENPNHTFGSFELIQAFTELARKKFPSESIEKCRKDPRNWCSISEVVETTSIVSEPKHYISIKDALKTITGC<br>ETKKFVKMLRNRLVKLGMEQGKDKIGEPSSFSLLESVDTLTEAQKENIRKGILEPSKLSFYNWRDIINKSLSDTLLTND<br>GNMIYCWLKSMASGVKRGLKPYIKNLRYDREAKSDATDCLKLILSNEEVENLQSFIRVMKAIVDPSRGEEEELYCVD<br>LLYSVWVKFASKVKFWKEIVESSLAAMTSCITDFENLIVMYTELNEQMKKYDCLSFTREEIALKAQELRFLEQHGNNI<br>VKFTNLIFFLCLACPWCMHYKSFELYLNKILETEPSRQLSPEAMKRLTSLGPVNLIKSRWFVDLCSENGLSPPDAEITK<br>HLDWLYRYCCALVSSNSEPLAATVNAMKPDEAVNTEQVLTKLRGILAKYNLEDSDLDFKWTISMIANSNFEVAKKLT<br>GRTTGERLPRSVRSKVIYEMIKLVGCSGMAVLQQLAFSTILNHNHEFFAVLAPKAQLGGHRDLLVQESGTKVVHATTE<br>MFSRTLLSTTNDDGLTNSHLKEAILNEGLAKMMLSRRNHGRPVSEMDDDGCLQFFRVFCISGDNTKWGPIHCCSLFS<br>GMMQQLLKDYPDWCNMYKLTFLKNLYRKVEIPSAAIQKILTTFKTRLASKYKLETMRENELRKLLNESIEIWSDQPM<br>IQFLIRTYLCKGDMAMTCYNHMGQGIHHATSSILTSVMADGIELIIREFCKRTFPELTVEVAHAGSSDDYAKCITLSGAI<br>SKSTFERYERTFWPQMCRLKNLMAGVGRCCQMKDSAKTLCGDTMLEFYSEFMLSFRITPAVIKFIHTGLINSSVTSPQ<br>SMVQACQVSGQQAMYNSVPLITNICFTVLRQQMFFNHTEFFMREYGPLVHGLPSSFCRLYIPVYSNLTGSSIAIEDSESI<br>SQDLKTLKDALPFLPNTKPLILSSELKDLVGSDDTKSTSSSHGSDKLTSGESSEGSSSNFRFELKRELTETESEYLKSVH<br>RDIDEASEMLVETRTKALFVGEEDYDGFPCFKRIKESNLIRSNFEFVKLLEEKPILRTFGLVRSVICAIIAGYYRSFSSEG<br>TEKTVKANLNRDENRVIEDPMIQLVPEKLRRELERLGLSRLSINELIGSGFSGLSLSEQIARRVVTMNCATEEYSSEIER<br>LKQTLSSRNVIHGLAGGIKELSLPIYTIFLKSYFFKDNVFFDHKDRWNTQHSTNYRDSTGKKLDGKIVTKFTVWLDRI<br>LSSTLSVSYKSQDFEPSLFNSSLKLVDIIRMEEGPTDLSILSAELDVFLSEFRSLAMQFSEPNRLKLKIVESRPHTKELDA<br>NKVIIVKSSIFNASEQVKIQNNPALIVGCMLSETIIMDVKPARVDYGGLMMDKHKVNCFYESIATVCNSITERSKALEA<br>SDESPDLDEVSTSANTLTMLCRLIQKQNSRLVNFYMIKPMATDTEPTVTDLVSYGTKEGRLLLLPEPNVEVSSFSVKY<br>WKIMQSISAIANLNLTQQEKTDLLCSFLHWKPNKSIAMQDCPLMKHEMSMLSEFQGSSLLATLSSEVMNIKNESTRH<br>QLEDLIDFIKDPNLLVTKKPFIGTTAIFRTWGEGQKNGRFTFSSGNGESTGIFIRGKLHIYLSLESEILLSEVEKQVLGWM<br>NRRRTDIVVKEQHDYFLNLLPTYPMVPRKHTDGYIRGVVINRNNPRMLGFTPAKYNSLVVRVKKRILTVKKSSKTCD<br>CSEPRLYWTPNAITIIYDELTESATYHEEVVKLRQSVSMIIEPEKKVEVPSVAYQDFKVVIGKIRLDSEALLSSTVLLHY<br>YLCHSPEEVRMELSSKSQRMIELLTGEGVIPGYRVSQKLYEDSRSIKKDLRVTPNLQLNIAGRLENYLNSTDARVSDW<br>SEVQQILDESGCSNIDVQLEQKTLEDRVSWRCLLSKISNRAPSMLTLRTLVSLVGSETIPNFLSGLLVHADVLDKLLRL

GKSAKNELSASSLDDRGLELLLSCTLYQLQVENKILHCQTYSTGSIFSLVTSKTFDSGNDMILGFTLDDEDFLSFSITVPI
PPLRKDASKREKAMAQGHALVAYNLFFDEVNNFQEVCSNTSLWRLQHFPDRSEIYMEFDYTKAGNTTLLKLLVSSGL
GMKDSILRLSPLIQFIATLVGRMLPEYLLEKAKEVKQSTNSCEFATEDDLLYLSEEELEPAKKEPEHGLFDLSF

>YP_010840879.1 glycoprotein precursor [Yezo virus]

MLIMQCRDFLALWLLASMCLSESGDGSLNTTVKGEAPSPTTPAASAVAPTNKTLPEKTTTVAPGNTTRVATRPTSSPAL
SRWLAGLYSRNYEDFHRRVATGMVRSADGSARMESNILGYSGNLSKGEQLTTTLIRAVDPRIADVFQVKRTVLAVKD
RVAGYSATAAYNYLGKMVYNVDEDGVVTVTGLSDLGNSGYTLHLKTQGPHVPADYLRPGCEKLHNCCEGPEKQVD
IFSLRSENIKVSSEPGSKYLSLAYLTGYLGFDARGCKAVVENHGSMYLLTENSVEQVNTVNSGKVYPIIHIFIDRTDPGE
NCAISMCNLIPIPGATGGLPVRQTKKQLIKVSLGGPEHSAFSRKLLSLRTQHNHAHQTPCNQPSHIMEIDQYKLHAPES
SFAHSKSSICNGTLISNKDLSGVKGCYVVARATVMHNCKQADMEPFESRGKCLVDNKLVSCEASESCIRVKVNDTTFI
KVKSNDYHNIIKCTKECLVPAPKGSLVLVTCPDGEDIVLHTNKVDLDCPLLSYLGKPAIYVCRATYRPRVFYALIIWVV
FGYSIGRIAFILTFLLLWIFNRLTRTIIFRMDKQKDTCSGCGEFVDHTVKWARHDSCNSGSCPYCNKLSSLSKLKEHAK
TCTSKVKELEQDEKVLDIALVPKWSRRLESTIKAFSKTLSRIQWVVVSCLLMYFLFHPVSGLSSNLKGKDFWDKSITE
LEVCQPVCVYEEEYCTCPIQESPTLHNHSIHHIVKRSLLSLPVAGAPRDLDIEASWGTVHIENSYQPSVGGNHISLSWQ
SQNRVGSKVVVAGKSEAILRLDPRSGLKWSITSPDSSESRNIFVSILEYAQEYETQFEYLTSNRKLGTWMHGVCTGTCP
EKCGCKSHTCSSQKWENTRNWRCNPTWCWNIGGCTCCGADIDKPFQDWILTKWSTDYARTTVIACVEFEHGSRQCD
LIDAGTTISLGGISVSFSGVTGITTMLPRQIALVHKAPGNHKDFDLVKPIGMIDGTDLCKLQACTHGPAGDFQIYNLNG
LVDNDVTNMNFWKTKTEAIKKDWMSWQGTVLQYYCNPGDWPTCVMSGAVNDNSGAFENLWKRSTKLHQQYFFH
SEKIDIRDGSPTLTLKGRPYMGGGQITALLSVSGLELESKTVLPKGVKLHITMCEGCYGCATGITCSVSITLDSPDRYGL
HLSSNTEGVIASETTLTCSAGQVNDFKLRLFSSLPEDKICLRVREAPKESEDASSCLSVKLQPPEEVVLEHRSVLTSTGN
STCGKGFLSCLGSNIAHFFGSIGTFLKDFFGSIWKGLVVIIPIVIILILLILFGPNLLGLIKVCISGRKSTIDRKVYYESGKR
ELERYRQIIKEEEELLRRNDKKK

>BDT56375.1 nucleoprotein, partial [Yezo virus]

MRSKRRHRLGITEEQEQEALSRKGAEHAPWVEDWVKGGEDRIFSLPSWGGWNKKTKKGLLLGGTAVA

>BDT53110.1 nucleoprotein [Yezo virus]

MARLIEAKTREELDKWIHNLDTTHFLGLNTKYTKSLALQPCEPGSCPEAYISFDGKKSDELDAILSGALVDSLKFAAPI
PECAWNLTNKIFTEGMAWFNANKDQKCMAWDKKYDTLRSTLPSPEDVAQYQIAARKWRKDIGYECSPNNGIMRGE
VTKVYIAPKKYVNDMILMVTDMRSKRRHRLGITEEQEQEALSRKGAEHAPWVEDWVKGGEDRIFSLPSWGGWNK
KTKKGLLLGGTAVAHLVQKKVITLKIFRSKVEKAGELLNNQQKMDELGIDVSQARLMHAHMEGCLKEAEGLIKDSV
TSAYVQQGSALDTAFSTYYWMWKADVTLANFGALNEMAFLYGQKPVGQKKLLDTLKGTAYKWGSTLANMCATG
DFNGERVHMHPGVFTPHRMSEMTAAIGAFPLSNPVKIKEGSASYRYLTNLKTSESNPAAKVVCELFNTHRRAHSDWN
SPTSIVPPEHYFHQSLLERLGPFCHVSLVRGNALRVNIVEEMGTGGNP

>BDT53109.1 glycoprotein precursor [Yezo virus]

MLIMQCRDFLALWLLASVCLSESGDGSLNTTVKGEAPSPTTPAASAVASTNKTLPEKTTTVAPGNTTRVATRPTSSPAL
SRWLAGLYSRNYEDFHRRVATGMVRSADGSARMDSNILGYSGNLSKGEQLTTTLIRAVDPRIADVFQVKRTVLAVKD
RVAGYSATAAYNYLGKMVYNVDEDGVVTVTGLSDLGNSGYTLHLKTQGPHVPADYLRPGCEKLHNCCEGPEKQVD
IFSLRSENIKVSSEPGSKYLSLAYLTGYLGFDARGCKAVVENHGSMYLLTENSVEQVNTVNNGKVYPIIHIFIDRTDPGE
NCAISMCNLIPIPGATGGLPVRQTKKQLIKVSLGGPEHSAFSRKLLSLRTQHNHAHQTPCNQPSHIMEIDQYKLHAPES
SFAHSKSSICNGTLISNKDLSGVKGCYVVARATVMHNCKQADMEPFESRGKCLVDNKLVSCEASESCIRVKVNDTTFI
KVKSNDYHNIIKCTKECLVPAPKGSLVLVTCPDGEDIVLHTNKVDLDCPLLSYLGKPAIYVCRATYRPRVFYALIIWVV
FGYSIGRIAFILTFLLLWIFNRLTRTIIFRMDKQKDTCSGCGEFVDHTVKWARHDSCNSGSCPYCNKLSSLSKLKEHAK
TCTSKVKELEQDEKVLDIALVPKWSRRLESTIKAFSKTLSRIQWVVVSCLLMYFLFHPVSGLSSNLKGKDFWDKSITE
LEVCQPVCVYEEEYCTCPIQEPPTLHNHSIHHIVKRSLLSLPVAGAPRDLDIEASWGTVHIENSYQPSVGGNHISLSWQ
SQNRVGSKVVVAGKSEAILRLDPRSGLKWSITSPDSSESRNIFVSILEYAQEYETQFEYLTSNRKLGTWMHGVCTGTCP
EKCGCKSHTCSSQKWENTRNWRCNPTWCWNIGGCTCCGADIDKPFQDWILTKWSTDYARTTVIACVEFEHGSRQCD
LIDAGTTISLGGISVSFSGVTGITTMLPRQIALVHKAPGNHKDFDLVKPIGMIDGTDLCKLQACTHGPAGDFQIYNLNG
LVDNDVTNMNFWKTKTEAIKKDWMSWQGTVLQYYCNPGDWPTCVMSGAVNDNSGAFENLWKRSTKLHQQYFFH
SEKIDIRDGSPTLTLKGRPYMGGGQITALLSVSGLELESKTVLPKGVKLHITMCEGCYGCATGITCSVSITLDSPDRYGL
HLSSNTEGVIASETTLTCSAGQVNDFKLRLFSSLPEDKICLRVREAPKESEDASSCLSVKLQPPEEVVLEHRSVLTSTGN
STCGKGFLSCLGSNIAHFFGSIGTFLKDFFGSIWKGLVVIIPIVIILILLILFGPNLLSLIKVCISGRKSTIDRKVYYESGKRE
LERYRQIIKEEEELLRRNDKKK

>BDT53108.1 RNA-dependent RNA-polymerase [Yezo virus]

MAGKLSKRPELTWEHRINNLFVASIVMPVDESFKVVRMPGDGKCFFYSVSHNMFGTIQGHTRVKWMVRDFAEKHW
DLLTEAKEFYRSREEYIRDLNRETYWGGSIEAEILSLRTNQPIIIWRAEDGYTITSASMWRGDSGKGEINLIQSHNNHFD
AAYPPELAGTTSGPAVSREDKTVLEAVGETIIDEDDESDTEMEVAKLEDTFLPTYESERIELLSKPAMPPVGPVRRLPAR
LEEMLVNEQTLPGKVGRHLNKLYSCNVSVLFINPGEALLIPSHSEVGGKVSISELGRLWLNPSKRIRKENLRTVIAISDQ
LMAYVDKGYVLRTFVPKTFISRHAGLVPPDLVRDMTLTATVILLSTFLYKSRMVEKRTFIRACLRDEGRISGKKVSRIL
NKLTPHTLYNDPLSVVRQICLAMFGEECGRLVDLFSCLGPVSYLSLSSLELDSLTRNKYYQVLEELSRADTEDFDFSSK
EISEIKATVDCCKALREASDKESCEQATGTVLQILGISRQELPRRAKDNRREQEKLVINKFFNDRAISKFVSLQGRAYSG
TNLSNILSYTHNLLMSKDKFKLTEDDVGQLELEQRKIRAMLAKEERVPIAIICSMMENRFNNLFKRLPPSCAQECEVLF
DSIRNAESYASAWRSALRLKGVAYEGMFALKYNCTYIPEDQKPTLSMIVQTYYPDKFVDFLDKTQKRPEIRDYTPDFS
ICQMIVEHAEGLVPPKVSSVQLQAHDLEESGVKLRRTKEKAFPLPEVPIKELRSLDRLKELLLGTGKQNQPVHLKTEK
YDESDCTVTSREIIAIEVGYQTDVDGKVFSDTHKWQEAIKLLSKLGIKALVHICADSSSTRLSDWWISPEDARLLKESI
SFLFHEMGENSPSDVTDIMVANISTTKVRAQLKSGSIVKTPVTTKDVAAAWNAKLEKIRVRPTGAVLTDGLMDTVKK
ALVEGVTMDRASCEKVIEEVVIKNMDEIISLVEETKNKHEVARNYVTANRLILGWLMEDIECCRCKACVAMVKDAIK
GIASEQESVKYLCSQIQLSEHLPCCHTDLPSVKSRSLFSTRCPDITTVRHMTGDGSTDDSELTELDKAIRLTLPGKTERE
RKIKRGLEQLIRLSMSDSHIPCIKLPTGQILINRHAFNPTKVSEKRAAKNDELRDSKLERLTKLLSKERLTGYSEKACKI
MNTLLEKTYLQGNSRCCLKPEWAQSVLMDLKTDRNEIDIATKLKETSEKRLNFIVNNDKIIPVSNSEIRQFIVDKSDTIL
NRKKNSKLFNLDCLIFKETVLEVLRRLNGTPYFDCIEIIATLTKLLLSFDWFQELVLYSKLCETFLQCCTEFNRSGIKVR
RVRHTSINLAIQLPSNKKENMKCCCYNLDFSRNKSTFLMNRRVAVLGAAYPYILIITTLQCLQHQRCLDELTKEHASLQ
RDILTANAKLLDLLQSTLCKANLGSFEQAALELRRHCKQTGNFLDHSSYDMIVNTLSGLSVVFGLALGDTLLLNSQPF
NKQIQNMRFTMLSGLSRLVSPLELGKKLSSSSRRIESGVSRLYLQLISYCCVEQVEQNSENWKQFDLCPNVFMPSLSIF
GMNTSGDRQLIFDIYLVHIYNKEMDNFDEGCIKVLEETAEKHMSWELSLMNDLEKRTKKHERHARLLLGLPTLPRHE
LPTEDAIPEDSFSTSSSTSAISSTRSFSSGKNIKTLFGRLKSEIKPFVVEDCLEVSRDELNDFKQVISDVGRGYSYEPNLQ
RLYKNVKTIIKENPNHTFGSFELIQAFTELARKKFPSESIEKCRKDPRNWCSISEVVETTSIVSEPKHYISIKDALKTITGC
ETKKFVKMLRNRLVKLGMEQGKDKIGEPSSFSLLESVDTLTEAQKENIRKGILEPSKLSFYNWRDIISKSLSDTLLTND
GNMIYCWLKSMASGVKRGLKPYIKNLRYDREAKSDATDCLKLILSNEEVENLQSFIRVMKAIVDPSRGEEEELYCVD
LLYSVWVKFASKVKFWKEIVESSLAAMTSCITDFENLIVMYTELNEQMKKYDCLSFTREEITLKAQELRFLEQHGNNI
VKFTNLIFFLCLACPWCMHYKSFELYLNKILETEPSRQLSPEAMKRLTSLGPVNLIKSRWFVDLCSENGLSPPDAETTK
HLDWLYRYCCALVSSNSEPLAATVNAMKPDEAVNTEQVLTKLRGILAKYNLEDSDLDFKWTISMIANSNFEVAKKLT
GRTTGERLPRSVRSKVIYEMIKLVGCSGMAVLQQLAFSTILNHNHEFFAVLAPKAQLGGHRDLLVQESGTKVVHATTE
MFSRTLLSTTNDDGLTNSHLKEAILNEGLAKMMLSRRNHGRPVSEIDDDGCLQFFRVFCISGDNTKWGPIHCCSLFSG
MMQQLLKDYPDWCNMYKLTFLKNLYRKVEIPSAAIQKILTTFKTRLASKYKLETMRENELRKLLNESIEIWSDQPMI
QFLIRTYLCKGDMAMTCYNHMGQGIHHATSSILTSVMADGIELIIREFCKRTFPELTVEVAHAGSSDDYAKCITLSGAIS
KSTFERYERTFWPQMCRLKNLMAGVGRCCQMKDSAKTLCGDTMLEFYSEFMLSFRITPAVIKFIHTGLINSSVTSPQS
MVQACQVSGQQAMYNSVPLITNICFTVLRQQMFFNHTEFFMREYGPLVHGLPSSFCRLYIPVYSNLTGSSIAVEDSESI
SQDLKTLKDALPFLPNTKPLILSSELKDLVGSDDTKSTSSSHGSDKLTSGESSEGSSSNFRFELKRELTETESEYLKSVH
RDIDEASEMLVETRTKALFVGEEDYDGFPCFKRIKESNLIRSNFEFVKLLEEKPILRTFGLVRSVICAIIAGYYRSFSSEG
TEKTVKANLNRDENRVIEDPMIQLVPEKLRRELERLGLSRLSINELIGSGFSGLSLSEQIARRVVTMNCATEEYSSEIER
LKQTLSSRNVIHGLAGGIKELSLPIYTIFLKSYFFKDNVFFDHKDRWNTQHSTNYRDSTGKKLDGKIVTKFTVWLDRI
LSSTLSVSYKSQDFEPSLFNSSLKLVDIIRMEEGPTDLSILSAELDVFLSEFRSLAMQFSEPNRLKLKIVESRPHTKELDA
NKVIIVKSSIFNASEQVKIQNNPALIVGCMLSETIIMDVKPARVDYGGLMMDKHKVNCFYESIATVCNSITERSKALEA
SDESPDLDEVSTSANTLTMLCRLIQKQNSRLVNFYMIKPMATDTEPTVTDLVSYGTKEGRLLLLPEPNVEVSSFSVKY
WKIMQSISAIANLNLTQQEKTDLLCSFLHWKPNKSIAMQDCPLMKHEMSMLSEFQGSSLLATLSSEVMNIKNESTRH
QLEDLIDFIKDPNLLVTKKPFIGTTAIFRTWGEGQKNGRFTFSSGNGESTGIFIRGKLHIYLSLESEILLSEVEKQVLGWM
NRRRTDIVVKEQHDYFLNLLPTYPMVPRKHTDGYIRGVVINRNNPRMLGFTPAKYNSLVVRVKKRILTVKKSSKTCD
CSEPRLYWTPNAITIIYDELTESATYHEEVVKLRQSVSMIIEPEKKVEVPSVAYQDFKVVIGKIRLDSEALLSSTVLLHY
YLCHSPEEVRMELSSKSQRMI
ELLTGEGVVPGYRVSQKLYEDSRSIKKDLWVTPNLQLNIAGRLENYLNSTDARVSDWSEVQQILDESGCSNIDVQLEQ
KTLEDRVSWRCLLSKISNRAPSMLTLRTLVSLVGSETIPNFLSGLLVHADVLDKLLRLGKSAKNELSASSLDDRGLELL
LSCTLYQLQVENKILHCQTYSTGSIFSLVTSKTFDSGNDMILGFTLDDEDFLSFSITVPIPPLRKDASKREKAMAQGHAL
VAYNLFFDEVNNFQEVCSNTSLWRLQHFPDRSEIYMEFDYTKAGNTTLLKLLVSSGLGMRDSILRLSPLIQFIATLVGR
MLPEYLLEKAKEIKQSTNSCEFATEDDLLYLSEEELEPAKKEPEHGLFDLSF

>BDT53107.1 nucleoprotein [Yezo virus]

MARLIEAKTREELDKWIHNLDTTHFLGLNTKYTKSLALQPCEPGSCPEAYISFDGKKSDELDAILSGALVDSLKFAAPI
PECAWNLTNKIFTEGMAWFNANKDQKCMAWDKKYDTLRSTLPSPEDVAQYQIAARKWRKDIGYECSPNNGIMRGE
VTKVYIAPKKYVNDMILMVTDMRSKRRHRLGITEEQEQEALSRKGAEHAPWVEDWVKGGEDRIFSLPSWGGWNK
KTKKGLLLGGTAVAHLVQKKVITLKIFRSKVEKAGELLNNQQKMDELGIDVSQARLMHAHMEGCLKEAEGLIKDSV
TSAYVQQGSALDTAFSTYYWMWKADVTLANFGALNEMAFLYGQKPVGQKKLLDTLKGTAYKWGSTLANMCATG
DFNGERVHMHPGVFTPHRMSEMTAAIGAFPLSNPVKIKEGSASYRYLTNLKTSESNPAAKVVCELFNTHRRAHSDWN
SPTSIVPPEHYFHQSLLERLGPFCHVSLVRGNALRVNIVEEMGTGGNP

>BDT53106.1 glycoprotein precursor [Yezo virus]

MLIMQCRDFLALWLLASVCLSESGDGSLNTTVKGEAPSPTTPAASAVAPTSKTLPEKTTTVAPGNTTRVATRPTSSPAL
SRWLAGLYSRNYEDFHRRVATGMVRSADGSARMESNILGYSGNLSKGEQLTTTLIRAVDPRIADVFQVKRTVLAVKD
RVAGYSATAAYNYLGKMVYNVDEDGVVTVTGLSDLGNSGYTLHLKTQGPHVPADYLRPGCEKLHNCCEGPEKQVD
IFSLRSENIKVSSEPGSKYLSLAYLTGYLGFDARGCKAVVENHGSMYLLTENSVEQVNTVNNGKVYPIIHIFIDRTDPGE
NCAISMCNLIPIPGATGGLPVRQTKKQLIKVSLGGPEHSAFSRKLLSLRTQHNHAHQTPCNQPSHIMEIDQYKLHAPES
SFAHSKSSICNGTLISNKDLSGVKGCYVVARATVMHNCKQADMEPFESRGKCLVDNKLVSCEASESCIRVKVNDTTFI
KVKSNDYHNIIKCTKECLVPAPKGSLVLVTCPDGEDIVLHTNKVDLDCPLLSYLGKPAIYVCRATYRPRVFYALIIWVV
FGYSIGRIAFILTFLLLWIFNRLTRTIIFRMDKQKDTCSGCGEFVDHTVKWARHDSCNSGSCPYCNKLSSLSKLKEHAK
TCTSKVKELEQDEKVLDIALVPKWSRRLESTIKAFSKTLSRIQWVVVSCLLMYFLFHPVSGLSSNLKGKDFWDKSITE
LEVCQPVCVYEEEYCTCPIQELPTLHNHSIHHIVKRSLLSLPVAGAPRDLDIEASWGTVHIENSYQPSVGGNHISLSWQ
SQNRVGSKVVVAGKSEAILRLDPRSGLKWSITSPDSSESRNIFVSILEYAQEYETQFEYLTSNRKLGTWMHGVCTGTCP
EKCGCKSHTCSSQKWENTRNWRCNPTWCWNIGGCTCCGADIDKPFQDWILTKWSTDYARTTVIACVEFEHGSRQCD
LIDAGTTISLGGISVSFSGVTGITTMLPRQIALVHKAPGNHKDFDLVKPIGMIDGTDLCKLQACTHGPAGDFQIYNLNG
LVDNDVTNMNFWKTKTEAIKKDWMSWQGTVLQYYCNPGDWPTCVMSGAVNDNSGAFENLWKRSTKLHQQYFFH
SEKIDIRDGSPALTLKGRPYMGGGQITALLSVSGLELESKTVLPKGVKLHITMCEGCYGCATGITCSVSITLDSPDRYGL
HLSSNTEGVIASETTLTCSAGQVNDFKLRLFSSLPEDKICLRVREAPKESEDASSCLSVKLQPPEEVVLEHRSVLTSTGN
STCGKGFLSCLGSNIAHFFGSIGTFLKDFFGSIWKGLVVIIPIVIILILLILFGPNLLSLIKVCISGRKSTIDRKVYYESGKRE
LERYRQIIKEEEELLRRNDKKK

>BDT53105.1 RNA-dependent RNA-polymerase [Yezo virus]
MAGKLSKRPELTWEHRINNLFVASIVMPVDESFKVVRMPGDGKCFFYSVSHNMFGTIQGHTRVKWMVRDFAEKHW
DLLTEAKEFYRNREEYIRDLNRETYWGGSIEAEILSLRTNQPIIIWRAEDGYTITSASMWRGDSGKGEINLIQSHNNHF
DAAYPPELAGTTSGPAVSREDKTVLEAVGETIIDEDDESDTEMEVAKLEDTFLPTYESERIELLSKPTMPPVGPVRRLPA
RLEEMLVNEQTLPGKVGRHLNKLYSCNVSVLFINPGEALLIPSHSEVGGKVSISELGRLWLNPSKRIRKENLRTVIAISD
QLMAYVDKGYVLRTFVPKTFISRHAGLVPPDLVRDMTLTATVILLSTFLYKSRMVEKRTFIRACLRDEGRISGKKVSRI
LNKLTPHTLYNDPLSVVRQICLAMFGEECGRLVDLFSCLGPVSYLSLSSLELDSLTRNKYYQVLEELSRADTEDFDFSS
KEISEIKATVDCCKALREASDKESCEQATGTVLQILGISRQELPRRAKDNRREQEKLVINKFFNDRAISKFVSLQGRAYS
GTNLSNILSYTHNLLMSKDKFKLTEDDVGQLELEQRKIRAMLAKEERVPIAIICSMMENRFNNLFKRLPPSCAQECEV
LFDSIRNAESYASAWRSALRLKGVAYEGMFALKYNCTYIPEDQKPTLSMIVQTYYPDKFVDFLDKTQKRPEIRDYTPD
FSICQMIVEHAEGLVPPKVSSVQLQAHDLEESGVKLRRTKEKAFPLPEVPIKELRSLDRLKELLLGTGKQNQPVHLKT
EKYDESDCTVTSREIIAIEVGYQTDVDGKVFSDTHKWQEAIKLLSKLGIKALVHICADSSSTRLSDWWISPEDARLLKE
SISFLFHEMGENSPSDVTDIMVANISTTKVRAQLKSGSIVKTPVTTKDVAAAWNAKLEKIRVRPTGAVLTDGLMDTVK
KALVEGVTMDRASCEKVIEEVVIKNMDEIISLVEETKNKHEVTRNYVTANRLILGWLMEDIECCRCKACVAMIKDAI
KSIASEQESVKYLCSQIQLSEHLPCCHTDLPSVKPRSLFSTRCPDITTVRHMTGDGSTDDSELTELDKAIRLTLPGKTER
ERKIKRGLEQLIRLSMSDSHIPCIKLPTGQILINRHAFNPTKVSEKRAAKNDEFRDSKLERLTKLLSKERLTGYSEKACK
IMNTLLEKTHLQGNSRCCLKPEWAQSVLMDLKTDRNEIDIATKLKETSEKRLNFIVNNDKIIPVSNSEIRQFIVDKSDTI
LNRKKNSKLFNLDCLIFKETVLEVLRRLNGTPYFDCIEIIATLTKLLLSFDWFQELVLYSKLCETFLQCCTEFNRSGIKV
RRVRHTSINLAIQLPSNKKENMKCCCYNLDFSRNKSTFLMNRRVAVLGAAYPYILIITTLQCLQHQRCLDELTKEHASL
QRDILTANAKLLDLLQSTLCKANLGSFEQAALELRRHCKQTGNFLDHSSYDMIVNTLSGLSVVFGLALGDTLLLNSQ
PFNKQIQNMRFTMLSGLSRLVSPLELGKKLSSSSRRIESGVSRLYLQLISYCCVEQVEQNSENWKQFDLCPNVFMPSLS
IFGMNTSGDRQLIFDIYLVHIYNKEMDNFDEGCIKVLEETAEKHMSWELSLMNDLEKRTKKHERHARLLLGLPTLPR
HELPTEDAIPEDSFSTSSSTSAISSTRSFSSGKNIKTLFGRLKSEIKPFVVEDCLEVSRDELNDFKQVISDVGRGYSYEPN
LQRLYKNVKTIIKENPNHTFGSFELIQAFTELARKKFPSESIEKCRKDPRNWCSISEVVETTSIVSEPKHYISIKDALKTIT
GCETKKFVKMLRNRLVKLGMEQGKDKIGEPSSFSLLESVDTLTEAQKENIRKGILEPSKLSFYNWRDIINKSLSDTLLT
NDGNMIYCWLKSMASGVKRGLKPYIKNLRYDREAKSDATDCLKLILSNEEVENLQSFIRVMKAIVDPSRGEEEELYC
VDLLYSVWVKFASKVKFWKEIVESSLAAMTSCITDFENLIVMYTELNEQMKKYDCLSFTREEIALKAQELRFLEQHG
NNIVKFTNLIFFLCLACPWCMHYKSFELYLNKILETEPSRQLSPEAMKRLTSLGPVNLIKSRWFVDLCSENGLSPPDAEI
TKHLDWLYRYCCALVSSNSEPLAATVNAMKPDEAVNTEQVLTKLRGILAKYNLEDSDLDFKWTISMIANSNFEVAKK
LTGRTTGERLPRSVRSKVIYEMIKLVGCSGMAVLQQLAFSTILNHNHEFFAVLAPKAQLGGHRDLLVQESGTKVVHAT
TEMFSRTLLSTTNDDGLTNSHLKEAILNEGLAKMMLSRRNHGRPVSEMDDDGCLQFFRVFCISGDNTKWGPIHCCSL
FSGMMQQLLKDYPDWCNMYKLTFLKNLYRKVEIPSAAIQKILTTFKTRLASKYKLETMRENELRKLLNESIEIWSDQP
MIQFLIRTYLCKGDMAMTCYNHMGQGIHHATSSILTSVMADGIELIIREFCKRTFPELTVEVAHAGSSDDYAKCITLSG
AVSKSTFERYERTFWPQMCRLKNLMAGVGRCCQMKDSAKTLCGDTMLEFYSEFMLSFRITPAVIKFIHTGLINSSVTS
PQSMVQACQVSGQQAMYNSVPLITNICFTVLRQQMFFNHTEFFMREYGPLVHGLPSSFCRLYIPVYSNLTGSSIAVEDS
ESISQDLKTLKDALPFLPNTKPLILSSELKDLVGSDDTKSTSSSHGSDKLTSGESSEGSSSNFRFELKRELTETESEYLKS
VHRDIDEASEMLVETRTKALFVGEEDYDGFPCFKRIKESNLIRSNFEFVKLLEEKPILRTFGLVRSVICAIIAGYYRSFSS
EGTEKTVKANLNRDENRVIEDPMIQLVPEKLRRELERLGLSRLSINELIGSGFSGLSLSEQIARRVVTMNCATEEYSSEI
ERLKQTLSSRNVIHGLAGGIKELSLPIYTIFLKSYFFKDNVFFDHKDRWNTQHSTNYRDSTGKKLDGKIVTKFTVWLD
RILSSTLSVSYKSQDFEPSLFNSSLKLVDIIRMEEGPTDLSILSAELDVFLSEFRSLAMQFSEPNRLKLKIVESRPHTKEL
DANKVIIVKSSIFNASEQVKIQNNPALIVGCMLSETIIMDVKPARVDYGGLMMDKHKVNCFYESIATVCNSITERSKAL
EASDESPDLDEVSTSANTLTMLCRLIQKQNSRLVNFYMIKPMATDTEPTVTDLVSYGTKEGRLLLLPEPNVEVSSFSVK
YWKIMQSISAIANLNLTQQEKTDLLCSFLHWKPNKSIAMQDCPLMKHEMSMLSEFQGSSLLATLSSEVMNIKNESTR

HQLEDLIDFIKDPNLLVTKKPFIGTTAIFRTWGEGQKNGRFTFSSGNGESTGIFIRGKLHIYLSLESEILLSEVEKQVLGW
MNRRRTDIVVKEQHDYFLNLLPTYPMVPRKHTDGYIRGVVINRNNPRMLGFTPAKYNSLVVRVKKRILTVKKSSKTC
DCSEPRLYWTPNAITIIYDELTESATYHEEVVKLRQSVSMIIEPEKKVEVPSVAYQDFKVVIGKIRLDSEALLSSTVLLH
YYLCHSPEEVRMELSSKSQRMIELLTGEGVIPGYRVSQKLYEDSRSIKKDLRVTPNLQLNIAGRLENYLNSTDARVSD
WSEVQQILDESGCSNIDVQLEQKTLEDRVSWRCLLSKISNRAPSMLTLRTLVSLVGSETIPNFLSGLLVHADVLDKLLR
LGKSAKNELSASSLDDRGLELLLSCTLYQLQVENKILHCQTYSTGSIFSLVTSKTFDSGNDMILGFTLDDEDFLSFSITV
PIPPLRKDASKKEKAMAQGHALVAYNLFFDEVNNFQEVCSNTSLWRLQHFPDRSEIYMEFDYTKAGNTTLLKLLVSS
GLGMRDSILRLSPLIQFIATLVGRMLPEYLLEKAKEVKQSTNSCEFATEDDLLYLSEEELEPARKEPEHGLFDLSF

>BDT53104.1 nucleoprotein [Yezo virus]

MARLIEAKTREELDKWIHNLDTTHFLGLNTKYTKSLALQPCEPGSCPEAYISFDGKKSDELDAILSGALVDSLKFAAPI
PECAWNLTNKIFTEGMAWFNANKDQKCMAWDKKYDTLRSTLPSPEDVAQYQIAARKWRKDIGYECSPNNGIMRGE
VTKVYIAPKKYVNDMILMVTDMRSKRRHRLGITEEQEQEALSRKGAEHAPWVEDWVKGGEDRIFSLPSWGGWNK
KTKKGLLLGGTAVAHLVQKKVITLKIFRSKVEKAGELLNNQQKMDELGIDVSQARLMHAHMEGCLKEAEGLIKDSV
TSAYVQQGSALDTAFSTYYWMWKADVTLANFGALNEMAFLYGQKPVGQKKLLDTLKGTAYKWGSTLANMCATG
DFNGERVHMHPGVFTPHRMSEMTAAIGAFPLSNPVKIKEGSASYRYLTNLKTSESNPAAKVVCELFNTHRRAHSDWN
SPTSIVPPEHYFHQSLLERLGPFCHVSLVRGNALRVNIVEEMGTGGNP

>BDT53103.1 glycoprotein precursor [Yezo virus]

MLIMQCRDFLALWLLASVCLSESGDGSLNTTVKGEAPSPTTPAASAVAPTNKTLPEKTTTVAPGNTTRVATRPTSSPAL
SRWLAGLYSRNYEDFHRRVATGMVRSADGSARMESNILGYSGNLSKGEQLTTTLIRAVDPRIADVFQVKRTVLAVKD
RVAGYSATAAYNYLGKMVYNVDEDGVVTVTGLSDLGNSGYTLHLKTQGPHVPADYLRPGCEKLHNCCEGPEKQVD
IFSLRSENIKVSSEPGSKYLSLAYLTGYLGFDARGCKAVVENHGSMYLLTENSVEQVNTVNNGKVYPIIHIFIDRTDPGE
NCAISMCNLIPIPGATGGLPVRQTKKQLIKVSLGGPEHSAFSRKLLSLRTQHNHAHQTPCNQPSHIMEIDQYKLHAPES
SFAHSKSSICNGTLISNKDLSGVKGCYVVARATVMHNCKQADMEPFESRGKCLVDNKLVSCEASESCIRVKVNDTTFI
KVKSNDYHNIIKCTKECLVPAPKGSLVLVTCPDGEDIVLHTNKVDLDCPLLSYLGKPAIYVCRATYRPRVFYALIIWVV
FGYSIGRIAFILTFLLLWIFNRLTRTIIFRMDKQKDTCSGCGEFVDHTVKWARHDSCNSGSCPYCNKLSSLSKLKEHAK
TCTSKVKELEQDEKVLDIALVPKWSRRLESTIKAFSKTLSRIQWVVVSCLLMYFLFHPVSGLSSNLKGKDFWDKITEL
EVCQPVCVYEEEYCTCPIQEPPTLHNHSIHHIVKRSLLSLPVAGAPRDLDIEASWGTVHIENSYQPSVGGNHISLSWQS
QNRVGSKVVVAGKSEAILRLDPRSGLKWSITSPDSSESRNIFVSILEYAQEYETQFEYLTSNRKLGTWMHGVCTGTCPE
KCGCKSHTCSSQKWENTRNWRCNPTWCWNIGGCTCCGADIDKPFQDWILTKWSTDYARTTVIACVEFEHGSRQCDL
IDAGTTISLGGISVSFSGVTGITTMLPRQIALVHKAPGNHKDFDLVKPIGMIDGTDLCKLQACTHGPAGDFQIYNLNGL
VDNDVTNMNFWKTKTEAIKKDWMSWQGTVLQYYCNPGDWPTCVMSGAVNDNSGAFENLWKRSTKLHQQYFFHS
EKIDIRDGSPTLTLKGRPYMGGGQITALLSVSGLELESKTVLPKGVKLHITMCEGCYGCATGITCSVSITLDSPDRYGL
HLSSNTEGVIASETTLTCSAVNDFKLRLFSSLPEDKICLRVREAPKESEDASSCLSVKLQPPEEVVLEHRSVLTSTGNST
CGKGFLSCLGSNIAHFFGSIGTFLKDFFGSIWKGLVVIIPIVIILILLILFGPNLLSLIKVCISGRKSTIDRKVYYESGKRELE
RYRQIIKEEEELLRRNDKKK

>BDT53102.1 RNA-dependent RNA-polymerase [Yezo virus]

MAGKLSKRPELTWEHRINNLFVASIVMPVDESFKVVRMPGDGKCFFYSVSHNMFGTIQGHTRVKWMVRDFAEKHW
DLLTEAKEFYRNREEYIRDLNRETYWGGSIEAEILSLRNNQPIIIWRAEDGYTITSASMWRGDSGKGEINLIQSHNNHF
DAAYPPELAGTTSGPAVSREDKTVLEAVGETIIDEDDESDTEMEVAKLEDTFLPTYESERIELLSKPTMPPVGPVRRLPA
RLEEMLVNEQTLPGKVGRHLNKLYSCNVSVLFINPGEALLIPSHSEVGGKVSISELGRLWLNPSKRIRRENLRTVIAISD
QLMAYVDKGYVLRTFVPKTFISRHAGLVPPDLVRDMTLTATVILLSTFLYKSRMVEKRTFIRACLRDEGRISGKKVSRI
LNKLTPHTLYNDPLSVVRQICLAMFGEECGRLVDLFSCLGPVSYLSLSSLELDSLTRNKYYQVLEELSRADTEDFDFSS
KEISEIKATVDCCKALREASDKESCEQATGTVLQILGISRQELPRRAKDNRREQEKLVINKFFNDRAISKFVSLQGRAYS
GTNLSNILSYTHNLLMSKDKFKLTEDDVGQLELEQRKIRAMLAKEERVPIAIICSMMENRFNNLFKRLPPSCAQECEV
LFDSIRNAESYASAWRSALRLKGVAYEGMFALKYNCTYIPEDQKPTLSMIVQTYYPDKFVDFLDKTQKRPEIRDYTPD
FSICQMIVEHAEGLVPPKVSSVQLQAHDLEESGVKLRRTKEKAFPLPEVPIKELRSLDRLKELLLGTGKQNQPVHLKT
EKYDESDCTVTSREIIAIEVGYQTDVDGKVFSDTHKWQEAIKLLSKLGIKALVHCADSSSTRLSDWWISPEDARLLKE
SISFLFHEMGENSPSDVTDIMVANISTTKVRAQLKSGSIVKTPVTTKDVAAAWNAKLEKIRVRPTGAVLTDGLMDTVK
KALVEGVTMDRASCEKVIEEVVIKNMDEIISLVEETKNKHEVTRNYVTANRLILGWLMEDIECCRCKACVAMIKDAI
KSIASEQESVKYLCSQIQLSEHLPCCHTDLPSVKPRSLFSTRCPDITTVRHMTGDGSTDDSELTELDKAIRLTLPGKTER
ERKIKRGLEQLIRLSMSDSHIPCIKLPTGQILINRHAFNPTKVSEKRAAKNDEFRDSKLERLTKLLSKERLTGYSEKACK
IMNTLLEKTHLQGNSRCCLKPEWAQSVLMDLKTDRNEIDIATKLKETSEKRLNFIVNNDKIIPVSNSEIRQFIVDKSDTI
LNRKKNSKLFNLDCLIFKETVLEVLRRLNGTPYFDCIEIIATLTKLLLSFDWFQELVLYSKLCETFLQCCTEFNRSGIKV
RRVRHTSINLAIQLPSNKKENMKCCCYNLDFSRNKSTFLMNRRVAVLGAAYPYILIITTLQCLQHQRCLDELTKEHASL
QRDILTANAKLLDLLQSTLCKANLGSFEQAALELRRHCKQTGNFLDHSSYDMIVNTLSGLSVVFGLALGDTLLLNSQ
PFNKQIQNMRFTMLSGLSRLVSPLELGKKLSSSSRRIESGVSRLYLQLISYCCVEQVEQNSENWKQFDLCPNVFMPSLS
IFGMNTSGDRQLIFDIYLVHIYNKEMDNFDEGCIKVLEETAEKHMSWELSLMNDLEKRTKKHERHARLLLGLPTLPR
HELPTEDAIPEDSFSTSSSTSAISSTRSFSSGKNIKTLFGRLKSEIKPFVVEDCLEVSRDELNDFKQVISDVGRGYSYEPN
LQRLYKNVKTIIKENPNHTFGSFELIQAFTELARKKFPSESIEKCRKDPRNWCSISEVVETTSIVSEPKHYISIKDALKTIT

GCETKKFVKMLRNRLVKLGMEQGKDKIGEPSSFSLLESVDTLTEAQKENIRKGILEPSKLSFYNWRDIINKSLSDTLLT
NDGNMIYCWLKSMASGVKRGLKPYIKNLRYDREAKSDATDCLKLILSNEEVENLQSFIRVMKAIVDPSRGEEEELYC
VDLLYSVWVKFASKVKFWKEIVESSLAAMTSCITDFENLIVMYTELNEQMKKYDCLSFTREEIALKAQELRFLEQHG
NNIVKFTNLIFFLCLACPWCMHYKSFELYLNKILETEPSRQLSPEAMKRLTSLGPVNLIKSRWFVDLCSENGLSPPDAEI
TKHLDWLYRYCCALVSSNSEPLAATVNAMKPDEAVNTEQVLTKLRGILAKYNLEDSDLDFKWTISMIANSNFEVAKK
LTGRTTGERLPRSVRSKVIYEMIKLVGCSGMAVLQQLAFSTILNHNHEFFAVLAPKAQLGGHRDLLVQESGTKVVHAT
TEMFSRTLLSTTNDDGLTNSHLKEAILNEGLAKMMLSRRNHGRPVSEMDDDGCLQFFRVLCISGDNTKWGPIHCCSL
FSGMMQQLLKDYPDWCNMYKLTFLKNLYRKVEIPSAAIQKILTTFKTRLASKYKLETMRENELRKLLNESIEIWSDQP
MIQFLIRTYLCK

>BDT53101.1 nucleoprotein [Yezo virus]
MARLIEAKTREELDKWIHNLDTTHFLGLNTKYTKSLALQPCEPGSCPEAYISFDGKKSDELDAILSGALVDSLKFAAPI
PECAWNLTNKIFTEGMAWFNANKDQKCMAWDKKYDTLRSTLPSPEDVAQYQIAARKWRKDIGYECSPNNGIMRGE
VTKVYIAPKKYVNDMILMVTDMRSKRRHRLGITEEQEQEALSRKGAKHAPWVEDWVKGGEDRIFSLPSWGGWNK
KTKKGLLLGGTAVAHLVQKKVITLKIFRSKVEKAGELLNNQQKMDELGIDVSQARLMHAHMEGCLKEAEGLIKDSV
TSAYVQQGSALDTAFSTYYWMWKADVTLANFGALNEMAFLYGQKPVGQKKLLDTLKGTAYKWGSTLANMCATG
DFNGERVHMHPGVFTPHRMSEMTAAIGAFPLSNPVKIKEGSASYRYLTNLKTSESNPAAKVVCELFNTHRRAHSDWN
SPTSIVPPEHYFHQSLLERLGPFCHVSLVRGNALRVNIVEEMGTGGNP

>BDT53100.1 glycoprotein precursor [Yezo virus]

MLIMQCRDFLALWLLASVCLSESGDGSLNTTVKGEAPSPTTPAASAVAPTNKTLPEKTTTVAPGNTTRVATRPTSSPAL
SRWLAGLYSRNYEDFHRRVATGMVRSADGSARMESNILGYSGNLSKGEQLTTTLIRAVDPRIADVFQVKRTVLAVKD
RVAGYSATAAYNYLGKMVYNVDEDGVVTVTGLSDLGNSGYTLHLKTQGPHVPADYLRPGCEKLHNCCEGPEKQVD
IFSLRSENIKVSSEPGSKYLSLAYLTGYLGFDARGCKAVVENHGSMYLLTENSVEQVNTVNSGKVYPIIHIFIDRTDPGE
NCAISMCNLIPIPGATGGLPVRQTKKQLIKVSLGGPEHSAFSRKLLSLRTQHNHAHQTPCNQPSHIMEIDQYKLHAPES
SFAHSKSSICNGTLISNKDLSGVKGCYVVARATVMHNCKQADMEPFESRGKCLVDNKLVSCEASESCIRVKVNDTTFI
KVKSNDYHNIIKCTKECLVPAPKGSLVLVTCPDGEDIVLHTNKVDLDCPLLSYLGKPAIYVCRATYRPRVFYALIIWVV
FGYSIGRIAFILTFLLLWIFNRLTRTIIFRMDKQKDTCSGCGEFVDHTVKWARHDSCNSGSCPYCNKLSSLSKLKEHAK
TCTSKVKELEQDEKVLDIALVPKWSRRLESTIKAFSKTLSRIQWVVVVSCLLMYFLFHPVSGLSSNLKGKDFWDKSITE
LEVCQPVCVYEEEYCTCPIQEPPTLHNHSIHHIVKRSLLSLPVAGAPRDLDIEASWGTVHIENSYQPSVGGNHISLSWQ
SQNRVGSKVVVAGKSEAILRLDPRSGLKWSITSPDSSESRNIFVSILEYAQEYETQFEYLTSNRKLGTWMHGVCTGTCP
EKCGCKSHTCSSQKWENTRNWRCNPTWCWNIGGCTCCGADIDKPFQDWILTKWSTDYARTTVIACVEFEHGSRQCD
LIDAGTTISLGGISVSFSGVTGITTMLPRQIALVHKAPGNHKDFDLVKPIGMIDGTDLCKLQACTHGPAGDFQIYNLNG
LVDNDVTNMNFWKTKTEAIKKDWMSWQGTVLQYYCNPGDWPTCVMSGAVNDNSGAFENLWKRSTKLHQQYFFH
SEKIDIRDGSPTLTLKGRPYMGGGQITALLSVSGLELESKTVLPKGVKLHITMCEGCYGCATGITCSVSITLDSPDRYGL
HLSSNTEGVIASETTLTCSAGQVNDFKLRLFCSLPEDKICLRVREAPKESEDASSCLSVKLQPPEEVVLEHRSVLTSTGN
STCGKGFLSCLGSNIAHFFGSIGTFLKDFFGSIWKGLVVIIPIVIILILLILFGPNLLSLIKVCISGRKSTIDRKVYYESGKRE
LERYRKIIKEEEELLRRNDKKK

>BDT53099.1 RNA-dependent RNA-polymerase [Yezo virus]
MAGKLSKRPELTWEHRINNLFVASIVMPVDESFKVVRMPGDGKCFFYSVSHNMFGTIQGHTRVKWMVRDFAEKHW
DLLTEAKEFYRNREEYIRDLNRETYWGGSIEAEILSLRTNQPIIIWRAEDGYTITSASMWRGDSGKGEINLIQSHNNHF
DAAYPPELAGTTSGPAVSREDKTVLEAVGETIIDEDDESDTEMEVAKLEDTFLPTYESERIELLSEPTMPPVGPVRRLPA
RLEEMLVNEQTLPGKVGRHLNKLYSCNVSVLFINPGEALLIPSHSEVGGKVSISELGRLWLNPSKRIRKENLRTVIAISD
QLMAYVDKGYVLRTFVPKTFISRHAGLVPPDLVRDMTLTATVILLSTFLYKSRMVEKRTFIRACLRDEGRISGKKVSRI
LNKLTPHTLYNDPLSVVRQICLAMFGEECGRLVDLFSCLGPVSYLSLSSLELDSLTRNKYYQVLEELSRADTEDFDFSS
KEISEIKATVDCCKALRESDKESCEQATGTVLQILGISRQELPRRAKDNRREQEKLVINKFFNDRAISKFVSLQGRAYSG
TNLSNILSYTHNLLMSKDKFKLTEDDVGQLELEQRKIRAMLAKEERVPIAIICSMMENRFNNLFKRLPPSCAQECEVLF
DSIRNAESYASAWRSALRLKGVAYEGMFALKYNCTYIPEDQKPTLSMIVQTYYPDKFVDFLDKTQKRPEIRDYTPDFS
ICQMIVEHAEGLVPPKVSSVQLQAHDLEESGVKLRRTKEKAFPLPEVPIKELRSLDRLKELLLGTGKQNQPVHLKTEK
YDESDCTVTSREIIAIEVGYQTDVDGKVFSDTHKWQEAIKLLSKLGIKALVHICADSSSTRLSDWWISPEDARLLKESI
SFLFHEMGENSPSDVTDIMVANISTTKVRAQLKSGSIVKTPVTTKDVAAAWNAKLEKIRVRPTGAVLTDGLMDTVKK
ALVEGVTMDRASCEKVIEEVVIKNMDEIISLVEETKNKHEVTRNYVTANRLILGWLMEDIECCRCKACVAMIKDAIKS
IASEQESVKYLCSQIQLSEHLPCCHTDLPSVKPRSLFSTRCPDITTVRHMTGDGSTDDSELTELDKAIRLTLPGKTERER
KIKRGLEQLIRLSMSDSHPCIKLPTGQILINRHAFNPTKVSEKRAAKNDEFRDSKLERLTKLLSKERLTGYSEKACKIM
NTLLEKTHLQGNSRCCLKPEWAQSVLMDLKTDRNEIDIATKLKETSEKRLNFIVNNDKIIPVSNSEIRQFIVDKSDTILD
RKKNSKLFNLDCLIFKETVLEVLRRLNGTPYFDCIEIIATLTKLLLSFDWFQELVLYSKLCETFLQCCTEFNRSGIKVRR
VRHTSINLAIQLPSNKKENMKCCCYNLDFSRNKSTFLMNRRVAVLGAAYPYILIITTLQCLQHQRCLDELTKEHASLQR
DILTANAKLLDLLQSTLCKANLGSFEQAALELRRHCKQTGNFLDHSSYDMIVNTLSGLSVVFGLALGDTLLLNSQPFN
KQIQNMRFTMLSGLSRLVSPLELGKKLSSSSRRIESGVSRLYLQLISYCCVEQVEQNSENWKQFDLCPNVFMPSLSIFG
MNTSGDRQLIFDIYLVHIYNKEMDNFDEGCIKVLEETAEKHMSWELSLMNDLEKRTKKHERHARLLLGLPTLPRHEL
PTEDAIPEDSFSTSSSTSAISSTRSFSSGKNIKTLFGRLKSEIKPFVVEDCLEVSRDELNDFKQVISDVGRGYSYEPNLQR
LYKNVKTIIKENPNHTFGSFELIQAFTELARKKFPSESIEKCRKDPRNWCSISEVVETTSIVSEPKHYISIKDALKTITGCE

TKKFVKMLRNRLVKLGMEQGKDKIGEPSSFSLLESVDTLTEAQKENIRKGILEPSKLSFYNWRDIINKSLSDTLLTNDG
NMIYCWLKSMASGVKRGLKPYIKNLRYDREAKSDATDCLKLILSNEEVENLQSFIRVMKAIVDSRGEEEELYCVDLL
YSVWVKFASKVKFWKEIVESSLAAMTSCITDFENLIVMYTELNEQMKKYDCLSFTREEIALKAQELRFLEQHGNNIV
KFTNLIFFLCLACPWCMHYKSFELYLNKILETEPSRQLSPEAMKRLTSLGPVNLIKSRWFVDLCSENGLSPPDAEITKH
LDWLYRYCCALVSSNSEPLAATVNAMKPDEAVNTEQVLTKLRGILAKYNLEDSDLDFKWTISMIANSNFEVAKKLTG
RTTGERLPRSVRSKVIYEMIKLVGCSGMAVLQQLAFSTILNHNHEFFAVLAPKAQLGGHRDLLVQESGTKVVHATTE
MFSRTLLSTTNDDGLTNSHLKEAILNEGLAKMMLSRRNHGRPVSEMDDDGCLQFFRVLCISGDNTKWGPIHCCSLFS
GMMQQLLKDYPDWCNMYKLTFLKNLYRKVEIPSAAIQKILTTFKTRLASKYKLETMRENELRKLLNESIEIWSDQPM
IQFLIRTYLCKGDMAMTCYNHMGQGIHHATSSILTSVMADGIELIIREFCKRTFPELTVEVAHAGSSDDYAKCITLSGAV
SSTFERYERTFWPQMCRLKNLMAGVGRCCQMKDSAKTLCGDTMLEFYSEFMLSFRITPAVIKFIHTGLIN

>WEY08193.1 polyprotein, partial [Yezo virus]

VKANLNRDENRVIEDPMIQLVPEKLRRELERLGLSRLSINELIGSGFSGLSLSEQIARRVVT

>UYR58269.1 nucleoprotein [Yezo virus]

MARLIEAKTREELDKWIHNLDTTHFLGLNTKYTKSLALQPCEPGSCPEAYISFDGKKSDELDAILSGALVDSLKFAAPI
PECAWNLTNKIFTEGMAWFNANKDQKCMAWDKKYDTLRSTLPSPEDVAQYQIAARKWRKDIGYECSPNNGIMRGE
VTKVYIAPKKYVNDMILMVTDMRSKRRHRLGITEEQEQEALSRKGAEHAPWVEDWVKGGEDRIFSLPSWGGWNK
KTKKGLLLGGTAVAHLVQKKVITLKIFRSKVEKAGELLNNQQKMDELGIDVSQARLMHAHMEGCLKEAEGLIKDSV
TSAYVQQGSALDTAFSTYYWMWKADVTLANFGALNEMAFLYGQKPVGQKKLLDTLKGTAYKWGSTLANMCATG
DFNGERVHMHPGVFTPHRMSEMTAAIGAFPLSNPVKIKEGSASYRYLTNLKTSESNPAAKVVCELFNTHRRAHSDWN
SPTSIVPPEHYFHQSLLERLGPFCHVSLVRGNALRVNIVEEIGTGGNP

>UYR58268.1 glycoprotein precursor [Yezo virus]

MLIMQYRDFLALWLLASVCLSESGDGSLNTTVKGEAPSPTTPAASAVAPTNKTLPEKTTTVAPGNTTRVATRPTSSPAL
SRWLAGLYSRNYEDFHRRVATGMVRSADGSARMESNILGYSGNLSKGEQLTTTLIRAVDPRIADVFQVKRTVLAVKD
RVAGYSATAAYNYLGKMVYNVDEDGVVTVTGLSDLGNSGYTLHLKTQGPHVPADYLRPGCEKLHNCCEGPEKQVD
IFSLRSENIKVSSEPGSKYLSLAYLTGYLGFDARGCKAVVENHGSMYLLTENSVEQVNTVNNGKVYPIIHIFIDRTDPGE
NCAISMCNLIPIPGATGGLPVRQTKKQLIKVSLGGPEHSAFSRKLLSLRTQHNHAHQTPCNQPSHIMEIDQYRLHAPES
SFAHSKSSICNGTLISNKDLSGVKGCYVVARATVMHNCKQADMEPFESRGKCLVDNKLVSCEASESCIRVKVNDTTFI
KVKSNDYHNIIKCTKECLVPAPKGSLVLVTCPDGEDIVLHTNKVDLDCPLLSYLGKPAIYVCRATYRPRVFYALIIWVV
FGYSIGRIAFILTFLLLWIFNRLTRTIIFRMDKQKDTCSGCGEFVDHTVKWARHDSCNSGSCPYCNKLSSLSKLKEHAK
TCTSKVKELEQDEKVLDIALVPKWSRRLESTIKAFSKTLSRIQWVVVVSCLLMYFLFHPVSGLSSNLKGKDFWDKSITE
LEVCQPVCVYEEEYCTCPIQESPTVHNHSIHHIVKRSLLSLPVAGAPRDLDIEASWGTVHIENSYQPSVGGNHISLSWQ
SQNRVGSKVVVAGKSEAILRLDPRSGLKWSITSPDSSESRNIFVSILEYAQEYETQFEYLTSNRKLGTWMHGVCTGTCP
EKCGCKSHTCSSQKWENTRNWRCNPTWCWNIGGCTCCGADIDKPFQDWILTKWSTEYARTTVIACVEFEHGSRQCD
LIDAGTTISLGGISVSFSGVTGITTMLPRQIALVHKAPGNHKDFDLVKPIGMIDGTDLCKLQACTHGPAGDFQIYNLNG
LVDNDVTNMNFWKTKTEAIKKDWMSWQGTVLQYYCNPGDWPTCVMSGAVNDNSGAFENLWKRSTKLHQQYFFH
SEKIDIRDGSPTLTLKGRPYMGGGQITALLSVSGLELESKTVLPKGVKLHITMCEGCYGCATGITCSVSITLDSPDRYGL
HLSSNTEGVIASETTLTCSAGQVNDFKLRLFSSLPEDKICLRVREAPKESEDASSCLSVKLQPPEEVVLEHRSVLTSTGN
STCGKGFLSCLGSNIAHFFGSIGTFLKDFFGSIWKGLVVIIPIVIILILLILFGPNLLSLIKVCISGRKSTIDRKVYYESGKRE
LERYRQIIKEEEELLRRNDKKK

>UYR58267.1 RNA-dependent RNA polymerase [Yezo virus]

MAGKLSKRPELTWEHRINNLFVASIVMPVDESFKVVRMPGDGKCFFYSVSHNMFGTIQGHTRVKWMVRDFAEKHW
DLLTEAKEFYRSREEYIRDLNRETYWGGSIEAEILSLRTNQPIIIWRAEDGYTITSASMWRGDSGKGEINLIQSHNNHFD
AAYPPELAGTTSGPTVSREDKTVLEAVGETIIDEDDESDTEMEVAKLEDTFLPTYESERIELLSKPTMPPVGPVRRLPAR
LEEMLVNEQTLPGKVGRHLNKLYSCNVSVLFINPGEALLIPSHSEVGGKVSISELGRLWLNPSKRIRKENLRTVIAISDQ
LMAYVDKGYVLRTFVPKTFISRHAGLVPPDLVRDMTLTATVILLSTFLYKSRMVEKRTFIRACLRDEGRISGKKVSRIL
NKLTPHTLYNDPLSVVRQICLAMFGEECGRLVDLFSCLGPVSYLSLSSLELDSLTRNKYYQVLEELSRADTEDFDFSSK
EISEIKATVDCCKALREASDKESCEQATGTVLQILGISRQEIPRRAKDNRREQEKLVINKFFNDRAISKFVSLQGRAYSG
TNLSNILSYTHNLLMSKDKFKLTEDDVGQLELEQRKIRAMLAKEERVPIAIICSMMENRFNNLFKRLPPSCAQECEVLF
DSIRNAESYASAWRSALRLKGVAYEGMFALKYNCTYIPEDQKPTLSMIVQTYYPDKFVDFLDKTQKRPEIRDYTPDFS
ICQMIVEHAEGLVPPKVSSVQLQAHDLEESGVKLRRTKEKAFPLPEVPIKELRSLDRLKELLLGTGKQNQPVHLKTEK
YDESDCTVTSREIIAIEVGYQTDVDGKVFSDTHKWQEAIKLLSKLGIKALVHICADSSSTRLSDWWISPEDARLLKESI
SFLFHEMGENSPSDVTDIMVANISTTKVRAQLKSGSIVKTPVTTKDVAAAWNAKLEKIRVRPTGAVLTDGLMDTVKK
ALVEGVTMDRASCEKVIEEVIIKNMDEIISLVEETKNKHEVTRNYVTANRLILGWLMEDIECCRCKACVAMIKDAIKGI
ASEQESVKYLCSQIQLSEHLPCCHTDLPSVKPRSLFSTRCPDITTVRHMTGDGSTDDSELTELDKAIRLTLPGKTERER
KIKRGLEQLIRLSMSDSHIPCIKLPTGQILINRHAFNPTKVSEKRAAKNDEFRDSKLERLTKLLSKERLTGYSEKACKIM
NTLLEKTHLQGNSRCCLKPEWAQSVLMDLKTDRNEIDIATKLKETSEKRLNFIVNNDKIIPVSNSEIRQFIVDKSDTINR
KKNSKLFNLDCLIFKETVLEVLRRLNGTPYFDCIEIIATLTKLLLSFDWFQELVLYSKLCETFLQCCTEFNRSGIKVRRV
RHTSINLAIQLPSNKKENMKCCCYNLDFSRNKSTFLMNRRVAVLGAAYPYILIITTLQCLQHQRCLDELTKEHASLQRD
ILTANAKLLDLLQSTLCKANLGSFEQAALELRRHCKQTGNFLDHSSYDMIVNTLAGLSVVFGLALGDTLLLNSQPFN
KQIQNMRFTMLSGLSRLASPLELGKKLSSSSRRIESGVSRLYLQLISYCCVEQVEQNSENWKQFDLCPNVFMPSLSIFG

MNTSGDRQLIFDIYLVHIYNKEMDNFDEGCIKVLEETAEKHMSWELSLMNDLEKRTKKHERHARLLLGLPTLPRHEL
PTEDAIPEDSFSTSSSTSAISSTRSFSSGKNIKTLFGRLKSEIKPFVVEDCLEVSRDELNDFKQVISDVGRGYSYEPNLQR
LYKNVKTIIKENPNHTFGSFELIQAFTELARKKFPSESIEKCRKDPRNWCSISEVVETTSIVSEPKHYISIKDALKTITGCE
TKKFVKMLRNRLVKLGMEQGKDKIGEPSSFSLLESVDTLTEAQKENIRKGILEPSKLSFYNWRDIINKSLSDTLLTNDG
NMIYCWLKSMASGVKRGLKPYIKNLRYDREAKSDATDCLKLILSNEEVENLQSFIRVMKAIVDPSRGEEEELYCVDLL
YSVWVKFASKVKFWKEIVESSLAAMTSCITDFENLIVMYTELNEQMKKYDCLSFTREEIALKAQELRFLEQHGNNIV
KFTNLIFFLCLACPWCMHYKSFELYLNKILETEPSRQLSPEAMKRLTSLGPVNLIKSKWFVDLCSENGLSPPDAEITKH
LDWLYRYCCALVSSNSEPLAATVNAMKPDEAVNTEQVLTKLRGILAKYNLEDSDLDFKWTISMIANSNFEVAKKLTG
RTTGERLPRSVRSKVIYEMIKLVGCSGMAVLQQLAFSTILNHNHEFFAVLAPKAQLGGHRDLLVQESGTKVVHATTE
MFSRTLLSTTNDDGLTNSHLKEAILNEGLAKMMLSRRNHGRPVSEIDDDGCMQFFRVFCISGDNTKWGPIHCCSLFSG
MMQQLLKDYPDWCNMYKLTFLKNLYRKVEIPSAAIQKILTTFKTRLASKYKLETMRENELRKLLNESIEIWSDQPMI
QFLIRTYLCKGDMAMTCYNHMGQGIHHATSSILTSVMADGIELIIREFCKRTFPELTVEVAHAGSSDDYAKCITLSGAIS
KSTFERYERTFWPQMCRLKNLMAGVGRCCQMKDSAKTLCGDTMLEFYSEFMLSFRITPAVIKFIHTGLIN

>UYR58265.1 glycoprotein precursor [Yezo virus]
MLIMQYRDFLALWLLASVCLSESGDGSLNTTVKGEAPSPTTPAVSAVAPTNKTLPEKTTTVAPGNTTRVATRPTSSPAL
SRWLAGLYSRNYEDFHRRVATGMVRSADGSARMESNILGYSGNLSKGEQLTTTLIRAVDPRIADVFQVKRTVLAVKD
RVAGYSATAAYNYLGKMVYNVDEDGVVTVTGLSDLGNSGYTLHLKTQGPHVPADYLRPGCEKLHNCCEGPEKQVD
IFSLRSENIKVSSEPGSKYLSLAYLTGYLGFDARGCKAVVENHGSMYLLTENSVEQVNTVNNGKVYPIIHIFIDRTDPGE
NCAISMCNLIPIPGATGGLPVRQTKKQLIKVSLGGPEHSAFSRKLLSLRTQHNHAHQTPCNQPSHIMEIDQYKLHAPES
SFAHSKSSICNGTLISNKDLSGVKGCYVVARATVMHNCKQADMEPFESRGKCLVDNKLVSCEASESCIRVKVNDTTFI
KVKSNDYHNIIKCTKECLVPAPKGSLVLVTCPDGEDIVLHTNKVDLDCPLLSYLGKPAIYVCRATYRPRVFYALIIWVV
FGYSIGRIAFILTFLLLWIFNRLTRTIIFRMDKQKDTCSGCGEFVDHTVKWARHDSCNSGSCPYCNKLSSLSKLKEHAK
TCTSKVKELEQDEKVLDIALVPKWSRRLESTIKAFSKTLSRIQWVMVSCLLMYFLFHPVSGLSSNLKGKDFWDKSITE
LEVCQPVCVYEEEYCTCPIQESPTLHNHSIHHIVKRSLLSLPVAGAPRDLDIEASWGTVHIENSYQPSVGGNHISLSWQ
SQNRVGSKVVVAGKSEAILRLDPRSGLKWSITSPDSSESRNIFVSILEYAQEYETQFEYLTSNRKLGTWMHGVCTGTCP
EKCGCKSHTCSSQKWENTRNWRCNPTWCWNIGGCTCCGADIDKPFQDWILTKWSTDYARTTVIACVEFEHGSRQCD
LIDAGTTISLGGISVSFSGVTGITTMLPRQIALVHKAPGNHKDFDLVKPIGMIDGTDLCKLQACTHGPAGDFQIYNLNG
LVDNDVTNMNFWKTKTEAIKKDWMSWQGTVLQYYCNPGDWPTCVMSGAVNDNSGAFENLWKRSTKLHQQYFFH
SEKIDIRDGSPTLTLKGRPYMGGGQITALLSVSGLELESKTVLPKGVKLHITMCEGCYGCATGITCSVSITLDSPDRYGL
HLSSNTEGVIASETTLTCSAGQVNDFKLRLFSSLPEDKICLRVREAPKESEDASSCLSVKLQPPEEVVLEHRSVLTSTGN
STCGKGFLSCLGSNIAHFFGSIGTFLKDFFGSIWKGLVVIIPIVIILILLILFGPNLLSLIKVCISGRKSTIDRKVYYESGKRE
LERYRQIIKEEEELLRRNDKKKIPCIKLPTGQILINRHAFNPTKVSEKRAAKNDEFRDSKLERLTKLLSKERLTGYSEKA
CKIMNTLLEKTHLQGNSRCCLKPEWAQSVLMDLKTDRNEIDIATKLKETSEKRLNFIVNNDKIIPVSNSEIRQFIVDKS
DTILNRKKNSKLFNLDCLIFKETVLEVLRRLNGTPYFDCIEIIATLTKLLLSFDWFQELVLYSKLCETFLQCCTEFNRSGI
KVRRVRHTSINLAIQLPSNKKENMKCCCYNLDFSRNKSTFLMNRRVAVLGAAYPYILIITTLQCLQHQRCLDELTKEH
ASLQRDILTANAKLLDLLQSTLCKANLGSFEQAALELRRHCKQTGNFLDHSSYDMIVNTLAGLSVVFGLALGDTLLL
NSQPFNKQIQNMRFTMLSGLSRLVSPLELGKKLSSSSRRIESGVSRLLQLISYCCVEQVEQNSENWKQFDLCPNVFMP
SLSIFGMNTSGDRQLIFDIYLVHIYNKEMDNFDEGCIKVLEETAEKHMSWELSLMNDLEKRTKKHERHARLLLGLPTL
PRHELPTEDAIPEDSFSTSSSTSAISSTRSFSSGKNIKTLFGRLKSEIKPFVVEDCLEVSRDELNDFKQVISDVGRGYSYE
PNLQRLYKNVKTIIKENPNHTFGSFELIQAFTELARKKFPSESIEKCRKDLRNWCSISEVVETTSIVSEPKHYISIKDALK
TITGCETKKFMKMLRNRLVKLGMEQGKDKIGEPSSFSLLESVDTLTEAQKENIRKGILEPSKLSFYNWRDIINKSLSDL
LTNDGNMIYCWLKSMASGVKRGLKPYIKNLRYDREAKSDATDCLKLILSNEEIENLQSFIRVMKAIVDPSRGEEEELY
CVDLLYSVWVKFASKVKFWKEIVESSLAAMTSCITDFENLIVMYTELNEQMKKYDCLSFTREEIALKAQELRFLEQH
GNNIVKFTNLIFFLCLACPWCMHYKSFELYLNKILETEPSKQLSPEAMKRLTSLGPVNLIKSRWFVDLCSENGLSPPDA
EITKHLDWLYRYCCALVSSNSEPLAATVNAMKPDEAVNTEQVLTKLRGILAKYNLEDSDLDFKWTISMIANSNFEVAK
KLTGRTTGERLPRSVRSKVIYEMIKLVGCSGMAVLQQLAFSTILNHNHEFFAVLAPKAQLGGHRDLLVQESGTKVVHA
TTEMFSRTLLSTTNDDGLTNSHLKEAILEGLAKMMLSRRNHGRPVSEMDDDGCLQFFRVFCISGDNTKWGPIHCCSL
FSGMMQQLLKDYPDWCNMYKLTFLKNLYRKVEIPSAAIQKILTTFKTRMASKYKLETMRENELRKLLNESIEIWSDQ
PMIQFLIRTYLCKGDMAMTCYNHMGQGIHHATSSILTSVMADGIELIIREFCKRTFPELTVEVAHAGSSDDYAKCITLSG
AISKSTFERYERTFWPQMCRLKNLMAGVGRCCQMKDSAKTLCGDTMLEFYSEFMLSFRITPAVIKFIHTGLINSSVTSP
QSMVQACQVSGQQAMYNSVPLITNICFTVLRQQMFFNHTEFFMREYGPLVHGLPSSFCRLYIPVYSNLTGSSIAVEDSE
SISQDLKTLKDALPFLPNTKPLILSSELKDLVGSDDTKSTSSSHGSDKLTSGESSEGSSSNFRFELKRELTETESEYLKSV
HRDIDEASEMLVETRTKALFVGEEDYDGFPCFKRIKESNLIRSNFEFVKLLEEKPILRTFGLVRSVICAIIAGYYRSFSSE
GTEKTVKANLNRDENRVIEDPMIQLVPEKLRRELERLGLSRLSINELIGSGFSGLSLSEQIARRVVTMNCATEEYSSEIE
RLKQTLSSRNVIHGLAGGIKELSLPIYTIFLKSYFFKDNVFFDHKDRWNTQHSTNYRDSTGKKLDGKIVTKFTVWLDR
ILSSTLSVSYKSQDFEPSLFNSSLKLVDIIRMEEGPTDLSILSAELDVFLSEFRSLAMQFSEPNRLKLKIVESRPHTKELD
ANKVIIVKSSIFNASEQVKIQNNPALIVGCMLSETIIMDVKPARVDYGGLMMDKHKVNCFYESIATVCNSITERSKALE
ASDESPDLDEVSTSANTLTMLCRLIQKQNSRLVNFYMIKPMATDTEPTVTDLVSYGTKEGRLLLLPEPNVEVSSFSVK
YWKIMQSISAIANLNLTQQEKTDLLCSFLHWKPNKSIAMQDCPLMKHEMSMLSEFQGSSLLATLSSEVMNIKNESTR
HQLEDLIDFIKDPNLLVTKKPFIGTTAIFRTWGEGQKNGRFTFSSGNGESTGIFIRGKLHIYLSLESEILLSEVEKQVLGW
MNRRRTDIVVKEQHDYFLNLLPTYPMVPRKHTDGYIRGVVINRNNPRMLGFTPAKYNSLVVRVKKRILTVKKSSKTC
DCSEPRLYWTPNAITIIYDELTESATYHEEVVKLRQSVSMIIEPEKKVEVPSVAYQDFKVVIGKIRLDSEALLSSTVLLH

YYLCHSPEEVRMELSSKSQRMIELLTGEGVIPGYRVSQKLYGDSRSIKKDLRVTPNLQLNIAGRLETYLNSTDARVSD<br>WSEVQQILDESGCSNIDVQLEQKTLEDRVSWRCLLSKISNRAPSMLTLRTLVSLVGSETIPNFLSGLLVHADVLDKLLR<br>LGKSAKNELSASSLDDRGLELLLSCTLYQLQVENKILHCQTYSTGSIFSLVTSKTFDSGNDMILGFTLDDEDFLSFSITV<br>PIPPLRKDASKREKAMAQGHALVAYNLFFDEVNNFQEVCSNTSLWRLQHFPDRSEIYMEFDYTKASNTTLLKLLVSSG<br>LGMRDSILRLSPLIQFIATLVGRMLPEYLLEKAKEVKQSTNSCEFATEDDLLYLSEEELEPAKKEPEHGLFDLSF
>UYR58263.1 nucleoprotein [Yezo virus]
MARLIEAKTREELDKWIHNLDTTHFLGLNTKYTKSLALQPCEPGSCPEAYISFDGKKSDELDAILSGALVDSLKFAAPI<br>PECAWNLTNKIFTEGMAWFNANKDQKCMAWDKKYDTLRSTLPSPEDVAQYQIAARKWRKDIGYECSPNNGIMRGE<br>VTKVYIAPKKYVNDMILMVTDMRSKRRHRLGITEEQEQEALSRKGAEHAPWVEDWVKGGEDRIFSLPSWGGWNK<br>KTKKGLLLGGTAVAHLVQKKVITLKIFRSKVEKAGELLNNQQKMDELGIDVSQARLMHAHMEGCLKEAEGLIKDSV<br>TSAYVQQGSALDTAFSTYYWMWKADVTLANFGALNEMAFLYGQKPVGQKKLLDTLKGTAYKWGSTLANMCATG<br>DFNGERVHMHPGVFTPHRMSEMTAAIGAFPLSNPVKIKEGSASYRYLTNLKTSESNPAAKVVCELFNTHRRAHSDWN<br>SPTSIVPPEHYFHQSLLERLGPFCHVSLVRGNALRVNIVEEIGTGGNP
>UYR58262.1 glycoprotein precursor [Yezo virus]

MLIMQYRDFLALWLLASVCLSESGDGSLNTTVKGEAPSPTTPAASAVAPTNKTLPEKTTTVAPGNTTRVATRPTSSPAL
SRWLAGLYSRNYEDFHRRVATGMVRSADGSARMESNILGYSGNLSKGEQLTTTLIRAVDPRIADVFQVKRTVLAVKD
RVAGYSATAAYNYLGKMVYNVDEDGVVTVTGLSDLGNSGYTLHLKTQGPHVPADYLRPGCEKLHNCCEGPEKQVD
IFSLRSENIKVSSEPGSKYLSLAYLTGYLGFDARGCKAVVENHGSMYLLTENSVEQVNTVNNGKVYPIIHIFIDRTDPGE
NCAISMCNLIPIPGATGGLPVRQTKKQLIKVSLGGPEHSAFSRKLLSLRTQHNHAHQTPCNQPSHIMEIDQYRLHAPES
SFAHSKSSICNGTLISNKDLSGVKGCYVVARATVMHNCKQADMEPFESRGKCLVDNKLVSCEASESCIRVKVNDTTFI
KVKSNDYHNIIKCTKECLVPAPKGSLVLVTCPDGEDIVLHTNKVDLDCPLLSYLGKPAIYVCRATYRPRVFYALIIWVV
FGYSIGRIAFILTFLLLWIFNRLTRTIIFRMDKQKDTCSGCGEFVDHTVKWARHDSCNSGSCPYCNKLSSLSKLKEHAK
TCTSKVKELEQDEKVLDIALVPKWSRRLESTIKAFSKTLSRIQWVVVSCLLMYFLFHPVSGLSSNLKGKDFWDKSITE
LEVCQPVCVYEEEYCTCPIQESPTVHNHSIHHIVKRSLLSLPVAGAPRDLDIEASWGTVHIENSYQPSVGGNHISLSWQ
SQNRVGSKVVVAGKSEAILRLDPRSGLKWSITSPDSSESRNIFVSILEYAQEYETQFEYLTSNRKLGTWMHGVCTGTCP
EKCGCKSHTCSSQKWENTRNWRCNPTWCWNIGGCTCCGADIDKPFQDWILTKWSTEYARTTVIACVEFEHGSRQCD
LIDAGTTISLGGISVSFSGVTGITTMLPRQIALVHKAPGNHKDFDLVKPIGMIDGTDLCKLQACTHGPAGDFQIYNLNG
LVDNDVTNMNFWKTKTEAIKKDWMSWQGTVLQYYCNPGDWPTCVMSGAVNDNSGAFENLWKRSTKLHQQYFFH
SEKIDIRDGSPTLTLKGRPYMGGGQITALLSVSGLELESKTVLPKGVKLHITMCEGCYGCATGITCSVSITLDSPDRYGL
HLSSNTEGVIASETTLTCSAGQVNDFKLRLFSSLPEDKICLRVREAPKESEDASSCLSVKLQPPEEVVLEHRSVLTSTGN
STCGKGFLSCLGSNIAHFFGSIGTFLKDFFGSIWKGLVVIIPIVIILILLILFGPNLLSLIKVCISGRKSTIDRKVYYESGKRE
LERYRQIIKEEEELLRRNDKKK
>UYR58261.1 RNA-dependent RNA polymerase [Yezo virus]
MAGKLSKRPELTWEHRINNLFVASIVMPVDESFKVVRMPGDGKCFFYSVSHNMFGTIQGHTRVKWMVRDFAEKHW
DLLTEAKEFYRSREEYIRDLNRETYWGGSIEAEILSLRTNQPIIIWRAEDGYTITSASMWRGDSGKGEINLIQSHNNHFD
AAYPPELAGTTSGPTVSREDKTVLEAVGETIIDEDDESDTEMEVAKLEDTFLPTYSERIELLSKPTMPPVGPVRRLPARL
EEMLVNEQTLPGKVGRHLNKLYSCNVSVLFINPGEALLIPSHSEVGGKVSISELGRLWLNPSKRIRKENLRTVIAISDQL
MAYVDKGYVLRTFVPKTFISRHAGLVPPDLVRDMTLTATVILLSTFLYKSRMVEKRTFIRACLRDEGRISGKKVSRILN
KLTPHTLYNDPLSVVRQICLAMFGEECGRLVDLFSCLGPVSYLSLSSLELDSLTRNKYYQVLEELSRADTEDFDFSSKE
ISEIKATVDCCKALREASDKESCEQATGTVLQILGISRQEIPRRAKDNRREQEKLVINKFFNDRAISKFVSLQGRAYSGT
NLSNILSYTHNLLMSKDKFKLTEDDVGQLELEQRKIRAMLAKEERVPIAIICSMMENRFNNLFKRLPPSCAQECEV
LFDSIRNAESYASAWRSALRLKGVAYEGMFALKYNCTYIPEDQKPTLSMIVQTYYPDKFVDFLDKTQKRPEIRDYTPD
FSICQMIVEHAEGLVPPKVSSVQLQAHDLEESGVKLRRTKEKAFPLPEVPIKELRSLDRLKELLLGTGKQNQPVHLKT
EKYDESDCTVTSREIIAIEVGYQTDVDGKVFSDTHKWQEAIKLLSKLGIKALVHCADSSSTRLSDWWISPEDARLLKE
SISFLFHEMGENSPSDVTDIMVANISTTKVRAQLKSGSIVKTPVTTKDVAAAWNAKLEKIRVRPTGAVLTDGLMDTVK
KALVEGVTMDRASCEKVIEEVIIKNMDEIISLVEETKNKHEVTRNYVTANRLILGWLMEDIECCRCKACVAMIKDAIK
GIASEQESVKYLCSQIQLSEHLPCCHTDLPSVKPRSLFSTRCPDITTVRHMTGDGSTDDSELTELDKAIRLTLPGKTERE
RKIKRGLEQLIRLSMSDSHIPCIKLPTGQILINRHAFNPTKVSEKRAAKNDEFRDSKLERLTKLLSKERLTGYSEKACKI
MNTLLEKTHLQGNSRCCLKPEWAQSVLMDLKTDRNEIDIATKLKETSEKRLNFIVNNDKIIPVSNSEIRQFIVDKSDTI
LNRKKNSKLFNLDCLIFKETVLEVLRRLNGTPYFDCIEIIATLTKLLLSFDWFQELVLYSKLCETFLQCCTEFNRSGIKV
RRVRHTSINLAIQLPSNKKENMKCCCYNLDFSRNKSTFLMNRRVAVLGAAYPYILIITTLQCLQHQRCLDELTKEHASL
QRDILTANAKLLDLLQSTLCKANLGSFEQAALELRRHCKQTGNFLDHSSYDMIVNTLAGLSVVFGLALGDTLLLNSQ
PFNKQIQNMRFTMLSGLSRLASPLELGKKLSSSSRRIESGVSRLYLQLISYCCVEQVEQNSENWKQFDLCPNVFMPSLS
IFGMNTSGDRQLIFDIYLVHIYNKEMDNFDEGCIKVLEETAEKHMSWELSLMNDLEKRTKKHERHARLLLGLPTLPR
HELPTEDAIPEDSFSTSSSTSAISSTRSFSSGKNIKTLFGRLKSEIKPFVVEDCLEVSRDELNDFKQVISDVGRGYSYEPN
LQRLYKNVKTIIKENPNHTFGSFELIQAFTELARKKFPSESIEKCRKDPRNWCSISEVVETTSIVSEPKHYISIKDALKTIT
GCETKKFVKMLRNRLVKLGMEQGKDKIGEPSSFSLLESVDTLTEAQKENIRKGILEPSKLSFYNWRDIINKSLSDTLLT
NDGNMIYCWLKSMASGVKRGLKPYIKNLRYDREAKSDATDCLKLILSNEEVENLQSFIRVMKAIVDPSRGEEEELYC
VDLLYSVWVKFASKVKFWKEIVESSLAAMTSCITDFENLIVMYTELNEQMKKYDCLSFTREEIALKAQELRFLEQHG
NNIVKFTNLIFFLCLACPWCMHYKSFELYLNKILETEPSRQLSPEAMKRLTSLGPVNLIKSKWFVDLCSENGLSPPDAE
ITKHLDWLYRYCCALVSSNSEPLAATVNAMKPDEAVNTEQVLTKLRGILAKYNLEDSDLDFKWTISMIANSNFEVAK
KLTGRTTGERLPRSVRSKVIYEMIKLVGCSGMAVLQLAFSTILNHNHEFFAVLAPKAQLGGHRDLLVQESGTKVV
HATTEMFSRTLLSTTNDDGLTNSHLKEAILNEGLAKMMLSRRNHGRPVSEIDDDGCMQFFRVFCISGDNTKWGPIHC
CSLFSGMMQQLLKDYPDWCNMYKLTFLKNLYRKVEIPSAAIQKILTTFKTRLASKYKLETMRENELRKLLNESIEIWS
DQPMIQFLIRTYLCKGDMAMTCYNHMGQGIHHATSSILTSVMADGIELIIREFCKRTFPELTVEVAHAGSSDDYAKCIT
LSGAISKSTFERYERTFWPQMCRLKNLMAGVGRCCQMKDSAKTLCGDTMLEFYSEFMLSFRITPAVIKFIHTGLIN
SSVTSPQSMVQACQVSGQQAMYNSVPLITNICFTVLRQQMFFNHTEFFMREYGPLVHGLPSSFCRLYIPVYSNLTGSSI
AVEDSESISQDLKTLKDALPFLPNTKPLILSSELKDLVGSDDTKSTSSSHGSDKLTSGESSEGSSSNFRFELKRELTETES
EYLKSVHRDIDEASEMLVETRTKALFVGEEDYDGFPCFKRIKESNLIRSNFEFVKLLEEKPILRTFGLVRSVICAIIAGYY
RSFSSEGTEKTVKANLNRDENRVIEDPMIQLVPEKLRRELERLGLSRLSINELIGSGFSGLSLSEQIARRVVTMNCATEE
YSSEIERLKQTLSSRNVIHGLAGGIKELSLPIYTIFLKSYFFKDNVFFDHKDRWNTQHSTNYRDSTGKKLDGKIVTKLA
VWLDRILSSTLSVSYKSQDFEPSLFNSSLKLVDIIRMEEGPTDLSILSAELDVFLSEFRSLAMQFSEPNRLKLKIVESRPH
TKELDANKVIIVKSSIFNASEQVKIQNNPALIVGCMLSETIIMDVKPARVDYGGLMMDKHKVNCFYESIATVCNSITER
SKALEASDESPDLDEVSTSANTLTMLCRLIQKQNSRLVNFYMIKPMATDTEPTVTDLVSYGTKEGRLLLLPEPNVEVS
SFSVKYWKIMQSISAIANLNLTQQEKTDLLCSFLHWKPNKSIAMQDCPLMKHEMSMLSEFQGSSLLATLSSEVMNIK
NESTRHQLEDLIDFIKDPNLLVTKKPFIGTTAIFRTWGEGQKNGRFTFSSGNGESTGIFIRGKLHIYLSLESEILLSEVEKQ

VLGWMNRRRTDIVVKEQHDYFLNLLPTYPMVPRKHTDGYIRGVVINRNNPRMLGFTPAKYNSLVVRVKKRILTVKK
SSKTCDCSEPRLYWTPNAITIIYDELTESATYHEEVVKLRQSVSMIIEPEKKVEVPSVAYQDFKVVIGKIRLDSEALLSST
VLLHYYLCHSPEEVRMELSSKSQRMIELLTGEGVIPGYRVSQKLYEDSRSIKKDLRVTPNLQLNIAGRLENYLNSTDA
RVSDWSEVQQILDESGCSNIDVQLEQKTLEDRVSWRCLLSKISNRAPSMLTLRTLVSLVGSETIPNFLSGLLVHADVLD
KLLRLGKSAKNELSASSLDDRGLELLLSCTLYQLQVENKILHCQTYSTGSIFSLVTSKTFDSGNDMILGFTLDDEDFLS
FSITVPIPPLRKDASKREKAMAQGHALVAYNLFFDEVNNFQEVCSNTSLWRLQHFPDRSEIYMEFDYTKAGNTTLLKL
LVSSGLGMRDSILRLSPLIQFIATLVGRMLPEYLLEKAKEVKQSTNSCEFATEDDLLYLSEEELEPAKKEPEHGLFDLSF

>UYR58260.1 nucleoprotein [Yezo virus]

MARLIEAKTREELDKWIHNLDTTHFLGLNTKYTKSLALQPCEPGSCPEAYISFDGKKSDELDAILSGALVDSLKFAAP
VPECAWNLTNKIFTEGMAWFNANKDQKCMAWDKKYDTLRSTLPSPEDVAQYQIAARKWRKDIGYECSPNNGIMRG
EVTKVYIAPKKYVNDMILMVTDMRSKRRHRLGITEEQEQEALSRKGAEHAPWVEDWVKGGEDRIFSLPSWGGWNK
KTKKGLLLGGTAVAHLVQKKVITLKIFRSKVEKAGELLNNQQKMDELGIDVSQARLMHAHMEGCLKEAEGLIKDSV
TSAYVQQGSALDTAFSTYYWMWKADVTLANFGALNEMAFLYGQKPVGQKKLLDTLKGTAYKWGSTLANMCATG
DFNGERVHMHPGVFTPHRMSEMTAAIGAFPLSNPVKIKEGSASYRYLTNLKTSESNPAAKVVCELFNTHRRAHSDWN
SPTSIVPPEHYFHQSLLERLGPFCHVSLVRGNALRVNIVEEMGTGGKP

>UYR58259.1 glycoprotein precursor [Yezo virus]

MLIMQCRDFLALWLLASVCLSESGDGSLNTTVKGEAPSPTTPAASAVAPTNKTLPEKTTTVAPGNTTRVATRPTSSPAL
SRWLAGLYSRNYEDFHRRVATGMVRSADGSARMESNILGYSGNLSKGEQLTTTLIRAVDPRIADVFQVKRTVLAVKD
RVAGYSATAAYNYLGKMVYNVDEDGVVTVTGLSDLGNSGYTLHLKTQGPHVPADYLRPGCEKLHNCCEGPEKQVD
IFSLRSENIKVSSEPGSKYLSLAYLTGYLGFDARGCKAVVENHGSMYLLTENSVEQVNTVNNGKVYPIIHIFIDRTDPGE
NCAISMCNLIPIPGATGGLPVRQTKKQLIKVSLGGPEHSFSRKLLSLRTQHNHAHQTPCNQPSHIMEIDQYKLHAPESS
FAHSKSSVCNGTLISNKDLSGVRGCYVVARATVMHNCKQADMEPFESRGKCLVDNKLVSCEASESCIRVKVNDTTFI
KVKSNDYHNIIKCTKECLVPAPKGSLVLVTCPDGEDIVLHTNKVDLDCPLLSYLGKPAIYVCRATYRPRVFYALIIWVV
FGYSIGRIAFILTFLLLWIFNRLTRTIIFRMDKQKDTCSGCGEFVDHTVKWARHDSCNSGSCPYCNKLSSLSKLKEHAK
TCTSKVKELEQDEKVLDIALVPKWSRRLESTIKAFSKTLSRIQWVVVVSCLLMYFLFHPVSGLSSNLKGKDFWDKSITE
LEVCQPVCVYEEEYCTCPIQESPTLHNHSIHHIVKRSLLSLPVAGAPRDLDIEASWGTVHIENSYQPSVGGNHISLSWQ
SQNRVGSKVVVAGKSEAILRLDPRSGLKWSITSPDSSESRNIFVSILEYAQEYETQFEYLTSNRKLGTWMHGVCTGTCP
EKCGCKSHTCSSQKWENTRNWRCNPTWCWNIGGCTCCGADIDKPFQDWILTKWSTDYARTTVIACVEFEHGSRQCD
LIDAGTTISLGGISVSFSGVTGITTMLPRQIALVHKAPGNHKDFDLVKPIGMIDGTDLCKLQACTHGPAGDFQIYNLNG
LVDNDVTNMNFWKTKTEAIKKDWMSWQGTVLQYYCNPGDWPTCVMSGAVNDNSGAFENLWKRSTKLHQQYFFH
SEKIDIRDGSPTLTLKGRPYMGGGQITALLSVSGLELESKTVLPKGVKLHITMCEGCYGCATGITCSVSITLDSPDRYGL
HLSSNTEGVIASETTLTCSAGQVNDFKLRLFSSLPEDKICLRVREAPKESEDASSCLSVKLQPPEEVVLEHRSVLTSTGN
STCGKGFLSCLGSNIAHFFGSIGTFLKDFFGSIWKGLVVIIPIVIILILLILFGPNLLSLIKVCISGRKSTIDRKVYYESGKRE
LERYRQIIKEEEELLRRNDKKK

>UYR58258.1 RNA-dependent RNA polymerase [Yezo virus]

MAGKLSKRPELTWEHRINNLFVASIVMPVDESFKVVRMPGDGKCFFYSVSHNMFGTIQGHTRVKWMVRDFAEKHW
DLLTEAKEFYRSREEYIRDLNRETYWGGSIEAEILSLRTNQPIIIWRAEDGYTITSASMWRGDSGKGEINLIQSHNNHFD
AAYPPELASTTSGPAVSREDKTVLEAVGETIIDEDDESDTEMEVAKLEDTFLPTYESERIELLSKPTMPPVGPVRRLPAR
LEEMLVNEQTLPGKVGRHLNKLYSCNVSVLFINPGEALLIPSHSEVGGKVSISELGRLWLNPSKRIRKENLRTVIAISDQ
LMAYVDKGYVLRTFVPKTFISRHAGLVPPDLVRDMTLTATVILLSTFLYKSRMVEKRTFIRACLRDEGRISGKKVSRIL
NKLTPHTLYNDPLSVVRQICLAMFGEECGRLVDLFSCLGPVSYLSLSSLELDSLTRNKYYQVLEELSRADTEDFDFSSK
EISEIKATVDCCKALREASDKESCEQATGTVLQILGISRQELPRRAKDNRREQEKLVINKFFNDRAISKFVSLQGRAYSG
TNLSNILSYTHNLLMSKDKFKLTEDDVGQLELEQRKIRAMLAKEERVPIAIICSMMENRFNNLFKRLPPSCAQECEV
LFDSIRNAESYASAWRSALRLKGVAYEGMFALKYNCTYIPEDQKPTLSMIVQTYYPDKFVDFLDKTQKRPEIRDYTPD
FSICQMIVEHAEGLVPPKVSSVQLQAHDLEESGVKLRRTKEKAFPLPEVPIKELRSLDRLKELLLGTGKQNQPVHLKT
EKYDESDCTVTSREIIAIEVGYQTDVDGKVFSDTHKWQEAIKLLSKLGIKALVHICADSSSTRLSDWWISPEDARLLKE
SISFLFHEMGENSPSDVTDIMVANISTTKVRAQLKSGSIVKTPVTTKDVAAAWNAKLEKIRVRPTGAVLTDGLMDTVK
KALVEGVTMDRASCEKVIEEVVIKNMDEIISLVEETKNKHEVTRNYVTANRLILGWLMEDIECCRCKDCVAMIKDAI
KGIASEQESVKYLCSQIQLSEHLPCCHTDLPSVKPRSLFSTRCPDITTVRHMTGDGSTDDSELTELDKAIRLTLPGKTER
ERKIKRGLEQLIRLSMSDSHIPCIKLPTGQILINRHAFNPTKVSEKRAAKNDEFRDSKLERLTKLLSKERLTGYSEKACK
IMNTLLEKTHLQGNSRCCLKPEWAQSVLMDLKTDRNEIDIATKLKETSEKRLNFIVNNDKIIPVSNSEIRQFIVDKSDTI
LNRKKNSKLFNLDCLIFKETVLEVLRRLNGTPYFDCIEIIATLTKLLLSFDWFQELVLYSKLCETFLQCCTEFNRSGIKV
RRVRHTSINLAIQLPSNKKENMKCCCYNLDFSRNKSTFLMNRRVAVLGAAYPYILIITTLQCLQHQRCLDELTKEHASL
QRDILTANAKLLDLLQSTLCKANLGSFEQAALELRRHCKQTGNFLDHSSYDMIVNTLAGLSVVFGLALGDTLLLNSQ
PFNKQIQNMRFTMLSGLSRLVSPLELGKKLSSSSRRIESGVSRLYLQLVSYCCVEQVEQNSENWKQFDLCPNVFMPSL
SIFGMNTSGDRQLIFDIYLVHIYNKEMDNFDEGCIKVLEETAEKHMSWELSLMNDLEKRTKKHERHARLLLGLPTLPR
HELPTEDAIPEDSFSTSSSTSAISSTRSFSSGKNIKTLFGRLKSEIKPFVVEDCLEVSRDELNDFKQVISDVGRGYSYEPN
LQRLYKNVKTIIKENPNHTFGSFELIQAFTELARKKFPSESIEKCRKDPRNWCSISEVVETTSIVSEPKHYISIKDALKTIT
GCETKKFVKMLRNRLVKLGMEQGKDKIGEPSSFSLLESVDTLTEAQKENIRKGILEPSKLSFYNWRDIINKSLSDTLLT
NDGNMIYCWLKSMASGVKRGLKPYIKNLRYDREAKSDATDCLKLILSNEEVENLQSFIRVMKAIVDPSRGEEEELYC
VDLLYSVWVKFASKVKFWKEIVESSLAAMTSCITDFENLIVMYTELNEQMKKYDCLSFTREEIALKAQELRFLEQHG
NNIVKFTNLIFFLCLACPWCMHYKSFELYLNKILETEPSKQLSPEAMKRLTSLGPVNLIKSRWFVDLCSENGLSPPDAEI
TKHLDWLYRYCCALVSSNSEPLAATVNAMKPDEAVNTEQVLTKLRGILAKYNLEDSDLDFKWTISMIANSNFEVAKK
LTGRTTGERLPRSVRSKVIYEMIKLVGCSGMAVLQQLAFSTILNHNHEFFAVLAPKAQLGGHRDLLVQESGTKVVHAT
TEMFSRTLLSTTNDDGLTNSHLKEAILNEGLAKMMLSRRNHGRPVSEMDDDGCLQFFRVFCISGDNTKWGPIHCCSL
FSGMMQQLLKDYPDWCNMYKLTFLKNLYRKVEIPSAAIQKILTTFKTRMASKYKLETMRENELRKLLNESIEIWSDQ
PMIQFLIRTYLCKGDMAMTCYNHMGQGIHHATSSILTSVMADGIELIIREFCKRTFPELTVEVAHAGSSDDYAKCITLSG

AISKSTFERYERTFWPQMCRLKNLMAGVGRCCQMKDSAKTLCGDTMLEFYSEFMLSFRITPAVIKFIHTGLINSSVTSP
QSMVQACQVSGQQAMYNSVPLITNICFTVLRQQMFFNHTEFFMREYGPLVHGLPSSFCRLYIPVYSNLTGSSIAVEDSE
SISQDLKTLKDALPFLPNTKPLILSSELKDLVGSDDTKSTSSSHGSDKLTSGESSEGSSSNFRFELKRELTETESEYLKSV
HRDIDEASEMLVETRTKALFVGEEDYDGFPCFKRIKESNLIRSNFEFVKLLEEKPILRTFGLVRSVICAIIAGYYRSFSSE
GTEKTVKANLNRDENRVIEDPMIQLVPEKLRRELERLGLSRLSINELIGSGFSGLSLSEQIARRVVTMNCATEEYSSEIE
RLKQTLSSRNVIHGLAGGIKELSLPIYTIFLKSYFFKDNVFFDHKDRWNTQHSTNYRDSTGKKLDGKIVTKFTVWLDR
ILSSTLSVSYKSQDFEPSLFNSSLKLVDIIRMEEGPTDLSILSAELDVFLSEFRSLAMQFSEPNRLKLKIVESRPHTKELD
ANKVIIVKSSIFNASEQVKIQNNPALIVGCMLSETIIMDVKPARVDYGGLMMDKHKVNCFYESIATVCNSITERSKALE
ASDESPDLDEVSTSANTLTMLCRLIQKQNSRLVNFYMIKPMATDTEPTVTDLVSYGTKEGRLLLLPEPNVEVSSFSVK
YWKIMQSISAIANLNLTQQEKTDLLCSFLHWKPNKSIAMQDCPLMKHEMSMLSEFQGSSLLATLSSEVMNIKNESTR
HQLEDLIDFIKDPNLLVTKKPFIGTTAIFRTWGEGQKNGRFTFSSGNGESTGIFIRGKLHIYLSLESEILLSEVEKQVLGW
MNRRRTDIVVKEQHDYFLNLLPTYPMVPRKHTDGYIRGVVINRNNPRMLGFTPAKYNSLVVRVKKRILTVKKSSKTC
DCSEPRLYWTPNAITIIYDELTESATYHEEVVKLRQSVSMIIEPEKKVEVPSVAYQDFKVVIGKIRLDSEALLSSTVLLH
YYLCHSPEEVRMELSSKSQRMIELLTGEGVIPGYRVSQKLYEDSRSIKKDLRVTPNLQLNIAGRLETYLNSTDARVSD
WSEVQQILDESGCSNIDVQLEQKTLEDRVSWRCLLSKISNRAPSMLTLRTLVSLVGSETIPNFLSGLLVHADVLDKLLR
LGKSAKNELSASSLDDRGLELLLSCTLYQLQVENKILHCQTYSTGSIFSLVTSKTFDSVNDMILGFTLDDEDFLSFSITV
PIPPLRKDASKREKAMAQGHALVAYNLFFDEVNNFQEVCSNTSLWRLQHFPDRSEIYMEFDYTKAGNTTLLKLLVSS
GLGMRDSILRLSPLIQFIATLVGRMLPEYLLEKAKEVKQSTNSCEFATEDDLLYLSEEELEPAKKEPEHGLFDLSF

>UYR58257.1 nucleoprotein [Yezo virus]

MARLIEAKTREELDKWIHNLDTTHFLGLNTKYTKSLALQPCEPGSCPEAYISFDGKKSDELDAILSGALV
DSLKFAAPIPECAWNLTNKIFTEGMAWFNANKDQKCMAWDKKYDTLRSTLPSPEDVAQYQIAARKWRKDI
GYECSPNNGIMRGEVTKVYIAPKKYVNDMILMVTDMRSKRRHRLGITEEQEQEALSRKGAEHAPWVEDWV
KGGEDRIFSLPSWGGWNKKTKKGLLLGGTAVAHLVQKKVITLKIFRSKVEKAGELLNNQQKMDELGIDVS
QARLMHAHMEGCLKEAEGLIKDSVTSAYVQQGSALDTAFSTYYWMWKADVTLANFGALNEMAFLYGQKPV
GQKKLLDTLKGTAYKWGSTLANMCATGDFNGERVHMHPGVFTPHRMSEMTAAIGAFPLSNPVKIKEGSAS
YRYLTNLKTSESNPAAKVVCELFNTHRRAHSDWNSPTSIVPPEHYFHQSLLERLGPFCHVSLVRGNALRV
NIVEEMGAGGTH

>UYR58256.1 glycoprotein precursor [Yezo virus]

MLIMQCRDFLALWLLASVCLSESGDGSLNTTVKGEAPSPTTPAASAVAPTNKTLPEKTTTVAPGNTTRVATRPTSSPAL
SRWLAGLYSRNYEDFHRRVATGMVRSADGSARMESNILGYSGNLSKGEQLTTTLIRAVDPRIADVFQVKRTVLAVKD
RVAGYSATAAYNYLGKMVYNVDEDGVVTVTGLSDLGNSGYTLHLKTQGPHVPADYLRPGCEKLHNCCEGPEKQVD
IFSLRSENIKVSSEPGSKYLSLAYLTGYLGFDARGCKAVVENHGSMYLLTENSVEQVNTVNNGKVYPIIHIFIDRTDPGE
NCAISMCNLIPIPGATGGLPVRQTKKQLIKVSLGGPEHSAFSRKLLSLRTQHNHAHQTPCNQPSHIMEIDQYKLHAPES
SFAHSKSSICNGTLISNKDLSGVKGCYVVARATVMHNCKQADMEPFESRGKCLVDNKLVSCEASESCIRVKVNDTTFI
KVKSNDYHNIIKCTKECLVPAPKGSLVLVTCPDGEDIVLHTNKVDLDCPLLSYLGKPAIYVCRATYRPRVFYALIIWVV
FGYSIGRIAFILTFLLLWIFNRLTRTIIFRMDKQKDTCSGCGEFVDHTVKWARHDSCNSGSCPYCNKLSSLSKLKEHAK
TCTSKVKELEQDEKVLDIALVPKWSRRLESTIKAFSKTLSRIQWVVVVSCLLMYFLFHPVSGLSSNLKGKDFWDKSITE
LEVCQPVCVYEEEYCTCPIQESPAVHNHSIHHIVKRSLLSLPVAGAPRDLDIEASWGTVHIENSYQPSVGGNHISLSWQ
SQNRVGSKVVVAGKSEAILRLDPRSGLKWSITSPDSSESRNIFVSILEYAQEYETQFEYLTSNRKLGTWMHGVCTGTCP
EKCGCKSHTCSSQKWENTRNWRCNPTWCWNIGGCTCCGADIDKPFQDWILTKWSTDYARTTVIACVEFEHGSRQCD
LIDAGTTISLGGISVSFSGVTGITTMLPRQIALVHKAPGNHKDFDLVKPIGMIDGTDLCKLQACTHGPAGDFQIYNLNG
LVDNDVTNMNFWKTKTEAIKKDWMSWQGTVLQYYCNPGDWPTCVMSGAVNDNSGAFENLWKRSTKLHQQYFFH
SEKIDIRDGSPTLTLKGRPYMGGGQITALLSVSGLELESKTVLPKGVKLHITMCEGCYGCATGITCSVSITLDSPDRYGL
HLSSNTEGVIASETTLTCSAGQVNDFKLRLFSSLPEDKICLRVREAPKESEDASSCLSVKLQPPEEVVLEHRSVLTSTGN
STCGKGFLSCLGSNIAHFFGSIGTFLKDFFGSIWKGLVVIIPIVIILILLILFGPNLLSLIKVCISGRKSTIDRKVYYESGKRE
LERYRQIIKEEEELLRRNDKKK

>UYR58255.1 RNA-dependent RNA polymerase [Yezo virus]

MAGKLSKRPELTWEHRINNLFVASIVMPVDESFKVVRMPGDGKCFFYSVSHNMFGTIQGHTRVKWMVRDFAEKHW
DLLTEAKEFYRSREEYIRDLNRETYWGGSIEAEILSLRTNQPIIIWRAEDGYTITSASMWRGDSGKGEINLIQSHNNHFD
AAYPPELAGTTSGPAVSREDKTVLEAVGETIIDEDDESDTEMEVAKLEDTFLPTYESERIELLSKPTMPPVGPVRRLPAR
LEEMLVNEQTLPGKVGRHLNKLYSCNVSVLFINPGEALLIPSHSEVGGKVSISELGRLWLNPSKRIRKENLRTVIAISDQ
LMAYVDKGYVLRTFVPKTFISRHAGLVPPDLVRDMTLTATVILLSTFLYKSRMVEKRTFIRACLRDEGRISGKKVSRIL
NKLTPHTLYNDPLSVVRQICLAMFGEECGRLVDLFSCLGPVSYLSLSSLELDSLTRNKYYQVLEELSRADTEDFDFSSK
EISEIKATVDCCKALREASDKESCEQATGTVLQILGISRQELPRRAKDNRREQEKLVINKFFNDRAISKFVSLQGRAYSG
TNLSNILSYTHNLLMSKDKFKLTEDDVGQLELEQRKIRAMLAKEERVPIAIICSMMENRFNNLFKRLPPSCAQECEV
LFDSIRNAESYASAWRSALRLKGVAYEGMFALKYNCTYIPEDQKPTLSMIVQTYYPDKFVDFLDKTQKRPEIRDYTPD
FSICQMIVEHAEGLVPPKVSSVQLQAHDLEESGVKLRRTKEKAFPLPEVPIKELRSLDRLKELLLGTGKQNQPVHLKT
EKYDESDCTVTSREIIAIEVGYQTDVDGKVFSDTHKWQEAIKLLSKLGIKALVHICADSSSTRLSDWWISPEDARLLKE
SISFLFHEMGENSPSDVTDIMVANISTTKVRAQLKSGSIVKTPVTTKDVAAAWNAKLEKIRVRPTGAVLTDGLMDTVK
KALVEGVTMDRASCEKVIEEVIIKNMDEIISLVEETKNKHEVTRNYVTANRLILGWLMEDIECCRCKACVAMIKDAIK

GIASEQESVKYLCSQIQLSEHLPCCHTDLPSVKPRSLFSTRCPDITTVRHMTGDGSTDDSELTELDKAIRLTLPGKTERE
RKIKRGLEQLIRLSMSDSHIPCIKLPTGQILINRHAFNPTKVSEKRAAKNDEFRDSKLERLTKLLSKERLTGYSEKACKI
MNTLLERTHLQGNSRCCLKPEWAQSVLMDLKTDRNEIDIATKLKETSEKRLNFIVNNDKIIPVSNSEIRQFIVDKSDTI
LNRKKNSKLFNLDCLIFKETVLEVLRRLNGTPYFDCIEIIATLTKLLLSFDWFQELVLYSKLCETFLQCCTEFNRSGIKV
RRVRHTSINLAIQLPSNKKENMKCCCYNLDFSRNKSTFLMNRRVAVLGAAYPYILIITTLQCLQHQRCLDELTKEHASL
QRDILTANAKLLDLLQSTLCKANLGSFEQAALELRRHCKQTGNFLDHSSYDMIVNTLAGLSVVFGLALGDTLLLNSQ
PFNKQIQNMRFTMLSGLSRLVSPLELGKKLSSSSRRIESGVSRLYLQLISYCCVEQVEQNSENWKQFDLCPNVFMPSLS
IFGMNTSGDRQLIFDIYLVHIYNKEMDNFDEGCIKVLEETAEKHMSWELSLMNDLEKRTKKHERHARLLLGLPTLPR
HELPTEDAIPEDSFSTSSSTSAISSTRSFSSGKNIKTLFGRLKSEIKPFVVEDCLEVSRDELNDFKQVISDVGRGYSYEPN
LQRLYKNVKTIIKENPNTFGSFELIQAFTELARKKFPSESIEKCRKDPRNWCSISEVVETTSIVSEPKHYISIKDALKTITG
CETKKFVKMLRNRLVKLGMEQGKDKIGEPSSFSLLESVDTLTEAQKENIRKGILEPSKLSFYNWRDIINKSLSDTLLTN
DGNMIYCWLKSMASGVKRGLKPYIKNLRYDREAKSDATDCLKLILSNEEVENLQSFIRVMKAIVDPSRGEEEELYCV
DLLYSVWVKFASKVKFWKEIVESSLAAMTSCITDFENLIVMYTELNEQMKKYDCLSFTREEIALKAQELRFLEQHGN
NIVKFTNLIFFLCLACPWCMHYKSFELYLNKILETEPSRQLSPEAMKRLTSLGPVNLIKSKWFVDLCSENGLSPPDAEIT
KHLDWLYRYCCALVSSNSEPLAATVNAMKPDEAVNTEQVLTKLRGILAKYNLEDSDLDFKWTISMIANSNFEVAKKL
TGRTTGERLPRSVRSKVIYEMIKLVGCSGMAVLQQLAFSTILNHNHEFFAVLAPKAQLGGHRDLLVQESGTKVVHATT
EMFSRTLLSTTNDDGLTNSHLKEAILNEGLAKMMLSRRNHGRPVSEMDDDGCLQFFRVFCISGDNTKWGPIHCCSLF
SGMMQQLLKDYPDWCNMYKLTFLKNLYRKVEIPSAAIQKILTTFKSRLASKYKLETMRENELRKLLNESIEIWSDQP
MIQFLIRTYLCKGDMAMTCYNHMGQGIHHATSSILTSVMADGIELIIREFCKRTFPELTVEVAHAGSSDDYAKCITLSG
AISKSTFERYERTFWPQMCRLKNLMAGVGRCCQMKDSAKTLCGDTMLEFYSEFMLSFRITPAVIKFIHTGLINSSVTSP
QSMVQACQVSGQQAMYNSVPLITNICFTVLRQQMFFNHTEFFMREYGPLVHGLPSSFCRLYIPVYSNLTGSSIAVEDSE
SISQDLKTLKDALPFLPNTKPLILSSELKDLVGSDDTKSTSSSHGSDKLTSGESSEGSSSNFRFELKRELTETESEYLKSV
HRDIDEASEMLVETRTKALFVGEEDYDGFPCFKRIKESNLIRSNFEFVKLLEEKPILRTFGLVRSVICAIIAGYYRSFSSE
GTEKTVKANLNRDENRVIEDPMIQLVPEKLRRELERLGLSRLSINELIGSGFSGLSLSEQIARRVVTMNCATEEYSSEIE
RLKQTLSSRNVIHGLAGGIKELSLPIYTIFLKSYFFKDNVFFDHKDRWNTQHSTNYRDSTGKKLDGKIVTKFTVWLDR
ILSSTLSVSYKSQDFEPSLFNSSLKLVDIIRMEEGPTDLSILSAELDVFLSEFRSLAMQFSEPNRLKLKIVESRPHTKELD
ANKVIIVKSSIFNASEQVKIQNNPALIVGCMLSETIIMDVKPARVDYGGLMMDKHKVNCFYESIATVCNSITERSKALE
ASDESPDLDEVSTSANTLTMLCRLIQKQNSRLVNFYMIKPMATDTEPTVTDLVSYGTKEGRLLLLPEPNVEVSSFSVK
YWKIMQSISAIANLNLTQQEKTDLLCSFLHWKPNKSIAMQDCPLMKHEMSMLSEFQGSSLLATLSSEVMNIKNESTR
HQLEDLIDFIKDPNLLVTKKPFIGTTAIFRTWGEGQKNGRFTFSSGNGESTGIFIRGKLHIYLSLESEILLSEVEKQVLGW
MNRRRTDIVVKEQHDYFLNLLPTYPMVPRKHTDGYIRGVVINRNNPRMLGFTPAKYNSLVVRVKKRILTVKKSSKTC
DCSEPRLYWTPNAITIIYDELTESATYHEEVVKLRQSVSMIIEPEKKVEIPSVAYQDFKVVIGKIRLDSEALLSSTVLLHY
YLCHSPEEVRMELSSKSQRMIELLTGEGVIPGYRVSQKLYEDSRSIKKDLRVTPNLQLNIAGRLENYLNSTDARVSDW
SEVQQILDESGCSNIDVQLEQKTLEDRVSWRCLLSKISNRAPSMLTLRTLVSLVGSETIPNFLSGLLVHADVLDKLLRL
GKSAKNELSASSLDDRGLELLLSCTLYQLQVENKILHCQTYSTGSIFSLVTSKTFDSGNDMILGFTLDDEDFLSFSITVPI
PPLRKDASKREKAMAQGHALVAYNLFFDEVNNFQEVCSNTSLWRLQHFPDRSEIYMEFDYTKAGNTTLLKLLVSSGL
GMRDSILRLSPLIQFIATLVGRMLPEYLLEKAKEIKQSTNSCEFATEDDLLYLSEEELEPAKKEPEHGLFDLSF

>UYR58254.1 nucleoprotein [Yezo virus]

MARLIEAKTREELDKWIHNLDTTHFLGLNTKYTKSLALQPCEPGSCPEAYISFDGKKSDELDAILSGALV
DSLKFAAPIPECAWNLTNKIFTEGMAWFNANKDQKCMAWDKKYDTLRSTLPSPEDVAQYQIAARKWRKDI
GYECSPNNGIMRGEVTKVYIAPKKYVNDMILMVTDMRSKRRHRLGITEEQEQEALSRKGAEHAPWVEDWV
KGGEDRIFSLPSWGGWNKKTKKGLLLGGTAVAHLVQKKVITLKIFRSKVEKAGELLNNQQKMDELGIDVS
QARLMHAHMEGCLKEAEGLIKDSVTSAYVQQGSALDTAFSTYYWMWKADVTLANFGALNEMAFLYGQKPV
GQKKLLDTLKGTAYKWGSTLANMCATGDFNGERVHMHPGVFTPHRMSEMTAAIGAFPLSNPVKIKEGSAS
YRYLTNLKTSESNPAAKVVCELFNTHRRAHSDWNSPTSIVPPEHYFHQSLLERLGPFCHVSLVRGNALRV
NIVEEMGTGGKP

>UYR58253.1 glycoprotein precursor [Yezo virus]
MLIMQCRDFLALWLLASVCLSESGDGSLNTTVKGEAPSPTTPAASAVAPTNKTLPEKTTTVAPGNTTRVA
TRPTSSPALSRWLAGLYSRNYEDFHRRVATGMVRSADGSARMESNILGYSGNLSKGEQLTTTLIRAVDPR
IADVFQVKRTVLAVKDRVAGYSATAAYNYLGKMVYNVDEDGVVTVTGLSDLGNSGYTLHLKTQGPHVPAD
YLRPGCEKLHNCCEGPEKQVDIFSLRSENIKVSSEPGSKYLSLAYLTGYLGFDARGCKAVVENHGSMYLL
TENSVEQVNTVNNGKVYPIIHIFIDRTDPGENCAISMCNLIPIPGATGGLPVRQTKKQLIKVSLGGPEHS
AFSRKLLSLRTQHNHAHQTPCNQPSHIMEIDQYKLHAPESSFAHSKSSICNGTLISNKDLSGVKGCYVVA
RATVMHNCKQADMEPFESRGKCLVDNKLVSCEASESCIRVKVNDTTFIKVKSNDYHNIIKCTKECLVPAP
KGSLVLVTCPDGEDIVLHTNKVDLDCPLLSYLGKPAIYVCRATYRPRVFYALIIWVVFGYSIGRIAFILT
FLLLWIFNRLTRTIIFRMDKQKDTCSGCGEFVDHTVKWARHDSCNSGSCPYCNKLSSLSKLKEHAKTCTS
KVKELEQDEKVLDIALVPKWSRRLESTIKAFSKTLSRIQWVVVVSCLLMYFLFHPVSGLSSNLKGKDFWDK
SITELEVCQPVCVYEEEYCTCPIQESPTLHNHSIHHIVKRSLLSLPVAGAPRDLDIEASWGTVHIENSYQ
PSVGGNHISLSWQSQNRVGSKVVVAGKSEAILRLDPRSGLKWSITSPDSSESRNIFVSILEYAQEYETQF
EYLTSNRKLGTWMHGVCTGTCPEKCGCKSHTCCSQKWENTRNWRCNPTWCWNIGGCTCCGADIDKPFQDW
ILTKWSTDYARTTVIACVEFEHGSRQCDLIDAGTTISLGGISVSFSGVTGITTMLPRQIALVHKAPGNHKDFDLVKPIGM
IDGTDLCKLQACTHGPAGDFQIYNLNGLVDNDVTNMNFWKTKTEAIKKDWMSWQGTVLQY
YCNPGDWPTCVMSGAVNDNSGAFENLWKRSTKLHQQYFFHSEKIDIRDGSPTLTLKGRPYMGGGQITALL
SVSGLELESKTVLPKGVKLHITMCEGCYGCATGITCSVSITLDSPDRYGLHLSSNTEGVIASETTLTCSA
GQVNDFKLRLFSSLPEDKICLRVREAPKESEDASSCLSVKLQPPEEVVLEHRSVLTSTGNSTCGKGFLSC
IGSNIAHFFGSIGTFLKDFFGSIWKGLVVIIPIVIILILLILFGPNLLSLIKVCISGRKSTIDRKVYYES
GRRELERYRQIIKEEEELLRRNDKKK

>UYR58252.1 RNA-dependent RNA polymerase [Yezo virus]
MAGKLSKRPELTWEHRINNLFVASIVMPVDESFKVVRMPGDGKCFFYSVSHNMFGTIQGHTRVKWMVRDF
AEKHWDLLTEAKEFYRSREEYIRDLNRETYWGGSIEAEILSLRTNQPIIIWRAEDGYTITSASMWRGDSG
KGEINLIQSHNNHFDAAYPPELAGTTSGPAVSREDKTVLEAVGETIIDEDDESDTEMEVAKLEDTFLPTY
ESERIELLSKPTMPPVGPVRRLPARLEEMLVNEQTLPGKVGRHLNKLYSCNVSVLFINPGEALLIPSHSE
VGGKVSISELGRLWLNPSKRIRKENLRTVIAISDQLMAYVDKGYVLRTFVPKTFISRHAGLVPPDLVRDM
TLTATVILLSTFLYKSRMVEKRTFIRACLRDEGRISGKKVSRILNKLTPHTLYNDPLSVVRQICLAMFGE
ECGRLVDLFSCLGPVSYLSLSSLELDSLTRNKYYQVLEELSRADTEDFDFSSKEISEIKATVDCCKALRE
ASDKDSCEQATGTVLQILGISRQELPRRARDNRREQEKLVINKFFNDRAISKFVSLQGRAYSGTNLSNIL
SYTHNLLMSKDKFKLTEDDVGQLELEQRKIRAMLAKEERVPIAIICSMMENRFNNLFKRLPPSCAQECEV
LFDSIRNAESYASAWRSALRLKGVAYEGMFALKYNCTYIPEDQKPTLSMIVQTYYPDKFVDFLDKTQKRP
EIRDYTPDFSICQMIVEHAEGLVPPKVSSVQLQAHDLEESGVKLRRTKEKAFPLPEVPIKELRSLDRLKE
LLLGTGKQNQPVHLKTEKYDESDCTVTSREIIAIEVGYQTDVDGKVFSDTHKWQEAIKLLSKLGIKALVH
ICADSSSTRLSDWWISPEDARLLKESISFLFHEMGENSPSDVTDIMVANISTTKVRAQLKSGSIVKTPVT
TKDVAAAWNAKLEKIRVRPTGAVLTDGLMDTVKKALVEGVTMDRASCEKVIEEVVIKNMDEIISLVEETK
NKHEVTRNYVTANRLILGWLMEDIECCRCKACVAMIKDAIKGIASEQESVKYLCSQIQLSEHLPCCHTDL
PSVKPRSLFSTRCPDITTVRHMTGDGSTDDSELTELDKAIRLTLPGKTERERKIKRGLEQLIRLSMSDSHIPCIKLPTGQI
LINRHAFNPTKVSEKRAAKNDEFRDSKLERLTKLLSKERLTGYSEKACKIMNTLLEKTH
LQGNSRCCLKPEWAQSVLMDLKTDRNEIDIATKLKETSEKRLNFIVNNDKIIPVSNSEIRQFIVDKSDTI
LNRKKNSKLFNLDCLIFKETVLEVLRRLNGTPYFDCIEIIATLTKLLLSFDWFQELVLYSKLCETFLQCC
TEFNRSGIKVRRVRHTSINLAIQLPSNKKENMKCCCYNLDFSRNKSTFLMNRRVAVLGAAYPYILIITTL
QCLQHQRCLDELTKEHASLQRDILTANAKLLDLLQSTLCKANLGSFEQAALELRRHCKQTGNFLDHSSYD
MIVNTLAGLSVVFGLALGDTLLLNSQPFNKQIQNMRFTMLSGLSRLVSPLELGKKLSSSSRRIESGVSRL
YLQLISYCCVEQVEQNSENWKQFDLCPNVFMPSLSIFGMNTSGDRQLIFDIYLVHIYNKEMDNFDEGCIK
VLEETAEKHMSWELSLMNDLEKRTKKHERHARLLLGLPTLPRHELPTEDAIPEDSFSTSSSTSAISSTRS
FSSGKNIKTLFGRLKSEIKPFVVEDCLEVSRDELNDFKQVISDVGRGYSYEPNLQRLYKNVKTIIKENPN

NEGLAKMMLSRRNHGRPVSEMDDDGCLQFFRVFCISGDNTKWGPIHCCSLFSGMMQQLLKDYPDWCNMYK
LTFLKNLYRKVEIPSAAIQKILTTFKTRLASKYKLETMRENELRKLLNESIEIWSDQPMIQFLIRTYLCK
GDMAMTCYNHMGQGIHHATSSILTSVMADGIELIIREFCKRTFPELTVEVAHAGSSDDYAKCITLSGAIS
KSTFERYERTFWPQMCRLKNLMAGVGRCCQMKDSAKTLCGDTMLEFYSEFMLSFRITPAVIKFIHTGLIN
SSVTSPQSMVQACQVSGQQAMYNSVPLITNICFTVLRQQMFFNHTEFFMREYGPLVHGLPSSFCRLYIPV
YSNLTGSSIAVEDSESISQDLKTLKDALPFLPNTKPLILSSELKDLVGSDDTKSTSSSHGSDKLTSGESSEGSSSNFRFELK
RELTETESEYLKSVHRDIDEASEMLVETRTKALFVGEEDYDGFPCFKRIKESNLIRSN
FEFVKLLEEKPILRTFGLVRSVICAIIAGYYRSFSSEGTEKTVKANLNRDENRVIEDPMIQLVPEKLRRE
LERLGLSRLSINELIGSGFSGLSLSEQIARRVVTMNCATEEYSSEIERLKQTLSSRNVIHGLAGGIKELS
LPIYTIFLKSYFFKDNVFFDHKDRWNTQHSTNYRDSTGKKLDGKIVTKFTVWLDRILSSTLSVSYKSQDF
EPSLFNSSLKLVDIIRMEEGPTDLSILSAELDVFLSEFRSLAMQFSEPNRLKLKIVESRPHTKELDANKV
IIVKSSIFNASEQVKIQNNPALIVGCMLSETIIMDVKPARVDYGGLMMDKHKVNCFYESIATVCNSITER
SKALEASDESPDLDEVSTSANTLTMLCRLIQKQNSRLVNFYMIKPMATDTEPTVTDLVSYGTKEGRLLLL
PEPNVEVSSFSVKYWKIMQSISAIANLNLTQQEKTDLLCSFLHWKPNKSIAMQDCPLMKHEMSMLSEFQG
SSLLATLSSEVMNIKNESTRHQLEDLIDFIKDPNLLVTKKPFIGTTAIFRTWGEGQKNGRFTFSSGNGES
TGIFIRGKLHIYLSLESEILLSEVEKQVLGWMNRRRTDIVVKEQHDYFLNLLPTYPMVPRKHTDGYIRGV
VINRNNPRMLGFTPAKYNSLVVRVKKRILTVKKSSKTCDCSEPRLYWTPNAITIIYDELTESATYHEEVV
KLRQSVSMIIEPEKKVEVPSVAYQDFKVVIGKIRLDSEALLSSTVLLHYYLCHSPEEVRMELSSKSQRMI
ELLTGEGVIPGYRVSQKLYEDSRSIKKDLRVTPNLQLNIAGRLENYLNSTDARVSDWSEVQQILDESGCS
NIDVQLEQKTLEDRVSWRCLLSKISNRAPSMLTLRTLVSLVGSETIPNFLSGLLVHADVLDKLLRLGKSA
KNELSASSLDDRGLELLLSCTLYQLQVENKILHCQTYSTGSIFSLVTSKTFDSGNDMILGFTLDDEDFLS
FSITVPIPPLRKDASKREKAMAQGHALVAYNLFFDEVNNFQEVCSNTSLWRLQHFPDRSEIYMEFDYTKA
GNTTLLKLLVSSGLGMRDSILRLSPLIQFIATLVGRMLPEYLLEKAKEVKQSTNSCEFATEDDLLYLSEE
ELEPAKKEPEHGLFDLSF

>WAK75218.1 MAG: hypothetical protein [Yezo virus]
MAGKLSKRPELTWEHRINNLFVASIVMPVDESFKVVRMPGDGKCFFYSVSHNMFGTIQGHTRVKWMVRDF
AEKHWDLLTEAKGFYRSREEYIRDLNRETYWGGSIEAEILSLRTNQPIIIWRAEDGYTITSASMWRGDSG
KGEINLIQSHNNHFDAAYPPELAGTTSGPAVSREDKTVLEAVGETIIDEDDESDTEMEVAKLEDTFLPTY
ESERIELLSKPTMPPVGPVRRLPARLEEMLVNEQTLPGKVGRHLNKLYSCNVSVLFINPGEALLIPSHSE
VGGKVSISELGRLWLNPSKRIRKENLRTVIAISDQLMAYVDKGYVLRTFVPKTFISRHAGLVPPDLVRDM
TLTATVILLSTFLYKSRMVEKRTFIRACLRDEGRISGKKVSRILNKLTPHTLYNDPLSVVRQICLAMFGE
ECGRLVDLFSCLGPVSYLSLSSLELDSLTRNKYYQVLEELSRADTEDFDFSSKEISEIKATVDCCKALRE
ASDKESCEQATGTVLQILGISRQELPRRAKDNRREQEKLVINKFFNDRAISKFVSLQGRAYSGTNLSNIL
SYTHNLLMSKDKFKLTEDDVGQLELEQRKIRAMLAKEERVPIAIICSMMENRFNNLFKRLPPSCAQECEV
LFDSIRNAESYASAWRSALRLKGVAYEGMFALKYNCTYIPEDQKPTLSMIVQTYYPDKFVDFLDKTQKRP
EIRDYTPDFSICQMIVEHAEGLVPPKVSSVQLQAHDLEESGVKLRRTKEKAFPLPEVPIKELRSLDRLKE
LLLGTGKQNQPVHLKTEKYDESDCTVTSREIIAIEVGYQTDVDGKVFSDTHKWQEAIKLLSKLGIKALVH
ICADSSSTRLSDWWISPEDARLLKESISFLFHEMGENSPSDVTDIMVANISTTKVRAQLKSGSIVKTPVT
TKDVAAAWNAKLEKIRVRPTGAVLTDGLMDTVKKALVEGVTMDRASCEKVIEEVIIKNMDEIISLVEETK
NKHEVTRNYVTANRLILGWLMEDIECCRCKACVAMIKDAIKGIASEQESVKYLCSQIQLSEHLPCCHTDL
PSVKPRSLFSTRCPDITTVRHMTGDGSTDDSELTELDKAIRLTLPGKTERERKIKRGLEQLIRLSMSDSH
IPCIKLPTGQILINRHAFNPTKVSEKRAAKNDEFRDSKLERLTKLLSKERLTGYSEKACKIMNTLLEKTH
LQGNSRCCLKPEWAQSVLMDLKTDRNEIDIATKLKETSEKRLNFIVNNDKIIPVSNSEIRQFIVDKSDTILNRKKNSKLF
NLDCLIFKETVLEVLRRLNGTPYFDCIEIIATLTKLLLSFDWFQELVLYSKLCETFLQCC
TEFNRSGIKVRRVRHTSINLAIQLPSNKKENMKCCCYNLDFSRNKSTFLMNRRVAVLGAAYPYILIITTL
QCLQHQRCLDELTKEHASLQRDILTANAKLLDLLQSTLCKANLGSFEQAALELRRHCKQTGNFLDHSSYD
MIVNTLAGLSVVFGLALGDTLLLNSQPFNKQIQNMRFTMLSGLSRLVSPLELGKKLSSSSRRIESGVSRL
YLQLISYCCVEQVEQNSENWKQFDLCPNVFMPSLSIFGMNTSGDRQLIFDIYLVHIYNKEMDNFDEGCIK
VLEETAEKHMSWELSLMNDLEKRTKKHERHARLLLGLPTLPRHELPTEDAIPEDSFSTSSSTSAISSTRS
FSSGKNIKTLFGRLKSEIKPFVVEDCLEVSRDELNDFKQVISDVGRGYSYEPNLQRLYKNVKTIIKENPN
HTFGSFELIQAFTELARKKFPSESIEKCRKDPRNWCSISEVVETTSIVSEPKHYISIKDALKTITGCETK
KFVKMLRNRLVKLGMEQGKDKIGEPSSFSLLESVDTLTEAQKENIRKGILEPSKLSFYNWRDIINKSLSD
TLLTNDGNMIYCWLKSMASGVKRGLKPYIKNLRYDREAKSDATDCLKLILSNEEVENLQSFIRVMKAIVD
PSRGEEEELYCVDLLYSVWVKFASKVKFWKEIVESSLAAMTSCITDFENLIVMYTELNEQMKKYDCLSFT
REEIALKAQELRFLEQHGNNIVKFTNLIFFLCLACPWCMHYKSFELYLNKILETEPSRQLSPEAMKRLTS
LGPVNLIKSKWFVDLCSENGLSPPDAEITKHLDWLYRYCCALVSSNSEPLAATVNAMKPDEAVNTEQVLT
KLRGILAKYNLEDSDLDFKWTISMIANSNFEVAKKLTGRTTGERLPRSVRSKVIYEMIKLVGCSGMAVLQ
QLAFSTILNHNHEFFAVLAPKAQLGGHRDLLVQESGTKVVHATTEMFSRTLLSTTNDDGLTNSHLKEAIL
NEGLAKMMLSRRNHGRPVSEMDDDGCLQFFRVFCISGDNTKWGPIHCCSLFSGMMQQLLKDYPDWCNMYK
LTFLKNLYRKVEIPSAAIQKILTTFKTRLASKYKLETMRENELRKLLNESIEIWSDQPMIQFLIRTYLCK
GDMAMTCYNHMGQGIHHATSSILTSVMADGIELIIREFCKRTFPELTVEVAHAGSSDDYAKCITLSGAIS

>WAK75149.1 MAG: hypothetical protein [Yezo virus]

YSNLTGSSIAVEDSESISQDLKTLKDALPFLPNTKPLILSSELKDLVGSDDTKSTSSSHGSDKLTSGESS
EGSSSNFRFELKRELTETESEYLKSVHRDIDEASEMLVETRTKALFVGEEDYDGFPCFKRIKESNLIRSN
FEFMKLLEEKPILRTFGLVRSVICAIIAGYYRSFSSEGTEKTVKANLNRDENRVIEDPMIQLVPEKLRRE
LERLGLSRLSINELIGSGFSGLSLSEQIARRVVTMNCATEEYSSEIERLKQTLSSRNVIHGLAGGIKELS
LPIYTIFLKSYFFKDNVFFDHKDRWNTQHSTNYRDSTGKKLDGKIVTKFTVWLDRILSSTLSVSYKSQDF
EPSLFNSSLKLVDIIRMEEGPTDLSILSAELDVFLSEFRSLAMQFSEPNRLKLKIVESRPHTKELDANKV
IIVKSSIFNASEQVKIQNNPALIVGCMLSETIIMDVKPARVDYGGLMMDKHKVNCFYESIATVCNSITER
SKALEASDESPDLDEVSTSANTLTMLCRLIQKQNSRLVNFYMIKPMATDTEPTVTDLVSYGTKEGRLLLL
PEPNVEVSSFSVKYWKIMQSISAIANLNLTQQEKTDLLCSFLHWKPNKSIAMQDCPLMKHEMSMLSEFQG
SSLLATLSSEVMNIKNESTRHQLEDLIDFIKDPNLLVTKKPFIGTTAIFRTWGEGQKNGRFTFSSGNGES
TGIFIRGKLHIYLSLESEILLSEVEKQVLGWMNRRRTDIVVKEQHDYFLNLLPTYPMVPRKHTDGYIRGV
VINRNNPRMLGFTPAKYNSLVVRVKKRILTVKKSSKTCDCSEPRLYWTPNAITIIYDELTESATYHEEVV
KLRQSVSMIIEPEKKVEVPSVAYQDFKVVIGKIRLDSEALLSSTVLLHYYLCHSPEEVRMELSSKSQRMI
ELLTGEGVIPGYRVSQKLYEDSRSIKKDLRVTPNLQLNIAGRLENYLNSTDARVSDWSEVQQILDESGCS
NIDVQLEQKTLEDRVSWRCLLSKISNRAPSMLTLRTLVSLVGSETIPNFLSGLLVHADVLDKLLRLGKSA
KNELSASSLDDRGLELLLSCTLYQLQVENKILHCQTYSTGSIFSLVTSKTFDSGNDMILGFTLDDEDFLS
FSITVPIPPLRKDASKREKAMAQGHALVAYNLFFDEVNNFQEVCSNTSLWRLQHFPDRSEIYMEFDYTKA
GNTTLLKLLVSSGLGMRDSILRLSPLIQFIATLVGRMLPEYLLEKAKEVKQSTNSCEFATEDDLLYLSEE
ELEPAKKEPEHGLFDLSF

>WAK75147.1 MAG: hypothetical protein [Yezo virus]
MAGKLSKRPELTWEHRINNLFVASIVMPVDESFKVVRMPGDGKCFFYSVSHNMFGTIQGHTRVKWMVRDF
AEKHWDLLTEAKEFYRNREEYIRDLNRETYWGGSIEAEILSLRTNQPIIIWRAEDGYTITSASMWRGDSG
KGEINLIQSHNNHFDAAYPPELAGTTSGPAVSREDKTVLEAVGETIIDEDDESDTEMEVAKLEDTFLPTY
ESERIELLSKPTMPPVGPVRRLPARLEEMLVNEQTLPGKVGRHLNKLYSCNVSVLFINPGEALLIPSHSE
VGGKVSISELGRLWLNPSKRIRKENLRTVIAISDQLMAYVDKGYVLRTFVPKTFISRHAGLVPPDLVRDM
TLTATVILLSTFLYKSRMVEKRTFIRACLRDEGRISGKKVSRILNKLTPHTLYNDPLSVVRQICLAMFGE
ECGRLVDLFSCLGPVSYLSLSSLELDSLTRNKYYQVLEELSRADTEDFDFSSKEISEIKATVDCCKALRE
ASDKESCEQATGTVLQILGISRQELPRRAKDNRREQEKLVINKFFNDRAISKFVSLQGRAYSGTNLSNIL
SYTHNLLMSKDKFKLTEDDVGQLELEQRKIRAMLAKEERVPIAIICSMMENRFNNLFKRLPPSCAQECEV
LFDSIRNAESYASAWRSALRLKGVAYEGMFALKYNCTYIPEDQKPTLSMIVQTYYPDKFVDFLDKTQKRP
EIRDYTPDFSICQMIVEHAEGLVPPKVSSVQLQAHDLEESGVKLRRTKEKAFPLPEVPIKELRSLDRLKE
LLLGTGKQNQPVHLKTEKYDESDCTVTSREIIAIEVGYQTDVDGKVFSDTHKWQEAIKLLSKLGIKALVH
ICADSSSTRLSDWWISPEDARLLKESISFLFHEMGENSPSDVTDIMVANISTTKVRAQLKSGSIVKTPVT
TKDVAAAWNAKLEKIRVRPTGAVLTDGLMDTVKKALVEGVTMDRASCEKVIEEVVIKNMDEIISLVEETK
NKHEVTRNYVTANRLILGWLMEDIECCRCKACVAMIKDAIKGIASEQESVKYLCSQIQLSEHLPCCHTDL
PSVKPRSLFSTRCPDITTVRHMTGDGSTDDSELTELDKAIRLTLPGKTERERKIKRGLEQLIRLSMSDSH
IPCIKLPTGQILINRHAFNPTKVSEKRTAKNDEFRDSKLERLTKLLSKERLTGYSEKACKIMNTLLEKTH
LQGNSRCCLKPEWAQSVLMDLKTDRNEIDIATKLKETSEKRLNFIVNNDKIIPVSNSEIRQFIVDKSDTI
LNRKKNSKLFNLDCLIFKETVLEVLRRLNGTPYFDCIEIIATLTKLLLSFDWFQELVLYSKLCETFLQCC
TEFNRSGIKVRRVRHTSINLAIQLPSNKKENMKCCCYNLDFSRNKSTFLMNRRVAVLGAAYPYILIITTL
QCLQHQRCLDELTKEHASLQRDILTANAKLLDLLQSTLCKANLGSFEQAALELRRHCKQTGNFLDHSSYD
MIVNTLAGLSVVFGLALGDTLLLNSQPFNKQIQNMRFTMLSGLSRLVSPLELGKKLSSSSRRIESGVSRL
YLQLISYCCVEQVEQNSENWKQFDLCPNVFMPSLSIFGMNTSGDRQLIFDIYLVHIYNKEMDNFDEGCIK
VLEETAEKHMSWELSLMNDLEKRTKKHERHARLLLGLPTLPRHELPTEDAIPEDSFSTSSSTSAISSTRS
FSSGKNIKTLFGRLKSEIKPFVVEDCLEVSRDELNDFKQVISDVGRGYSYEPNLQRLYKNVKTIIKENPN
HTFGSFELIQAFTELARKKFPSESIEKCRKDPRNWCSISEVVETTSIVSEPKHYISIKDALKTITGCETK
KFVKMLRNRLVKLGMEQGKDKIGEPSSFSLLESVDTLTEAQKENIRKGILEPSKLSFYNWRDIINKSLSD
TLLTNDGNMIYCWLKSMASGVKRGLKPYIKNLRYDREAKSDATDCLKLILSNEEVENLQSFIRVMKAIVD
PSRGEEEELYCVDLLYSVWVKFASKVKFWKEIVESSLAAMTSCITDFENLIVMYTELNEQMKKYDCLSFT
REEIALKAQELRFLEQHGNNIVKFTNLIFFLCLACPWCMHYKSFELYLNKILETEPSKQLSPEAMKRLTS
LGPVNLIKSKWFVDLCSENGLSPPDVEITKHLDWLYRYCCALVSSNSEPLAATVNAMKPDEAVNTEQVLT
KLRGILAKYNLEDSDLDFKWTISMIANSNFEVAKKLTGRTTGERLPRSVRSKVIYEMIKLVGCSGMAVLQ

QLAFSTILNHNHEFFAVLAPKAQLGGHRDLLVQESGTKVVHATTEMFSRTLLSTTNDDGLTNSHLKEAIL<br>NEGLAKMMLSRRNHGRPVSEMDDDGCLQFFRVFCISGDNTKWGPIHCCSLFSGMMQQLLKDYPDWCNMYK<br>LTFLKNLYRKVEIPSAAIQKILTTFKTRLASKYKLETMRENELRKLLNESIEIWSDQPMIQFLIRTYLCK<br>GDMAMTCYNHMGQGIHHATSSILTSVMADGIELIIREFCKRTFPELTVEVAHAGSSDDYAKCITLSGAIS<br>KSTFERYERTFWPQMCRLKNLMAGVGRCCQMKDSAKTLCGDTMLEFYSEFMLSFRITPAVIKFIHTGLIN<br>SSVTSPQSMVQACQVSGQQAMYNSVPLITNICFTVLRQQMFFNHTEFFMREYGPLVHGLPSSFCRLYIPV<br>YSNLTGSSIAVEDSESISQDLKTLKDALPFLPNTKPLILSSELKDLVGSDDTKSTSSSHGSDKLTSGESS<br>EGSSSNFRFELKRELTETESEYLKSVHRDIDEASEMLVETRTKALFVGEEDYDGFPCFKRIKESNLIRSN<br>FEFVKLLEEKPILRTFGLVRSVICAIIAGYYRSFSSEGTEKTVKANLNRDENRVIEDPMIQLVPEKLRRE<br>LERLGLSRLSINELIGSGFSGLSLSEQIARRVVTMNCATEEYSSEIERLKQTLSSRNVIHGLAGGIKELS<br>LPIYTIFLKSYFFKDNVFFDHKDRWNTQHSTNYRDSTGKKLDGKIVTKFTVWLDRILSSTLSVSYKSQDF<br>EPSLFNSSLKLVDIIRMEEGPTDLSILSAELDVFLSEFRSLAMQFSEPNRLKLKIVESRPHTKELDANKV<br>IIVKSSIFNASEQVKIQNNPALIVGCMLSETIIMDVKPARVDYGGLMMDKHKVNCFYESIATVCNSITER<br>SKALEASDESPDLDEVSTSANTLTMLCRLIQKQNSRLVNFYMIKPMATDTEPTVTDLVSYGTKEGRLLLL<br>PEPNVEVSSFSVKYWKIMQSISAIANLNLTQQEKTDLLCSFLHWKPNKSIAMQDCPLMKHEMSMLSEFQG<br>SSLLATLSSEVMNIKNESTRHQLEDLIDFIKDPNLLVTKKPFIGTTAIFRTWGEGQKNGRFTFSSGNGES<br>TGIFIRGKLHIYLSLESEILLSEVEKQVLGWMNRRRTDIVVKEQHDYFLNLLPTYPMVPRKHTDGYIRGV<br>VINRNNPRMLGFTPAKYNSLVVRVKKRILTVKKSSKTCDCSEPRLYWTPNAITIIYDELTESATYHEEVV<br>KLRQSVSMIIEPEKKVEVPSVAYQDFKVVIGKIRLDSEALLSSTVLLHYYLCHSPEEVRMELSSKSQRMI<br>ELLTGEGVIPGYRVSQKLYEDSRSIKKDLRVTPNLQLNIAGRLENYLNSTDARVSDWSEVQQILDESGCS<br>NIDVQLEQKTLEDRVSWRCLLSKISNRAPSMLTLRTLVSLVGSETIPNFLSGLLVHADVLDKLLRLGKSA<br>KNELSASSLDDRGLELLLSCTLYQLQVENKILHCQTYSTGSIFSLVTSKTFDSGNDMILGFTLDDEDFLS<br>FSITVPIPPLRKDASKREKAMAQGHALVAYNLFFDEVNNFQEVCSNTSLWRLQHFPDRSEIYMEFDYTKA<br>GNTTLLKLLVSSGLGMRDSILRLSPLIQFIATLVGRMLPEYLLEKAKEVKQSTNSCEFATEDDLLYLSEE<br>ELEPAKKEPEHGLFDLSF
>BCU83584.1 nucleoprotein [Yezo virus]
MARLIEAKTREELDKWIHNLDTTHFLGLNTKYTKSLALQPCEPGSCPEAYISFDGKKSDELDAILSGALV<br>DSLKFAAPIPECAWNLTNKIFTEGMAWFNANKDQKCMAWDKKYDTLRSTLPSPEDVAQYQIAARKWRKDI<br>GYECSPNNGIMRGEVTKVYIAPKKYVNDMILMVTDMRSKRRHRLGITEEQEQEALSRKGAEHAPWVEDWV<br>KGGEDRIFSLPSWGGWNKKTKKGLLLGGTAVAHLVQKKVITLKIFRSKVEKAGELLNNQQKMDELGIDVS<br>QARLMHAHMEGCLKEAEGLIKDSVTSAYVQQGSALDTAFSTYYWMWKADVTLANFGALNEMAFLYGQKPV<br>GQKKLLDTLKGTAYKWGSTLANMCATGDFNGERVHMHPGVFTPHRMSEMTAAIGAFPLSNPVKIKEGSAS<br>YRYLTNLKTSESNPAAKVVCELFNTHRRAHSDWNSPTSIVPPEHYFHQSLLERLGPFCHVSLVRGNALRV<br>NIVEEMGAGGTH
>BCU83583.1 nucleoprotein [Yezo virus]
MARLIEAKTREELDKWIHNLDTTHFLGLNTKYTKSLALQPCEPGSCPEAYISFDGKKSDELDAILSGALV<br>DSLKFAAPIPECAWNLTNKIFTEGMAWFNANKDQKCMAWDKKYDTLRSTLPSPEDVAQYQIAARKWRKDI<br>GYECSPNNGIMRGEVTKVYIAPKKYVNDMILMVTDMRSKRRHRLGITEEQEQEALSRKGAEHAPWVEDWV<br>KGGEDRIFSLPSWGGWNKKTKKGLLLGGTAVAHLVQKKVITLKIFRSKVEKAGELLNNQQKMDELGIDVS<br>QARLMHAHMEGCLKEAEGLIKDSVTSAYVQQGSALDTAFSTYYWMWKADVTLANFGALNEMAFLYGQKPV<br>GQKKLLDTLKGTAYKWGSTLANMCATGDFNGERVHMHPGVFTPHRMSEMTAAIGAFPLSNPVKIKEGSAS<br>YRYLTNLKTSESNPAAKVVCELFNTHRRAHSDWNSPTSIVPPEHYFHQSLLERLGPFCHVSLVRGNALRV<br>NIVEEMGTRGNP
>BCU83582.1 nucleoprotein [Yezo virus]
MARLIEAKTREELDKWIHNLDTTHFLGLNTKYTKSLALQPCEPGSCPEAYISFDGKKSDELDAILSGALV<br>DSLKFAAPIPECAWNLTNKIFTEGMAWFNANKDQKCMAWDKKYDTLRSTLPSPEDVAQYQIAARKWRKDI<br>GYECSPNNGIMRGEVTKVYIAPKKYVNDMILMVTDMRSKRRHRLGITEEQEQEALSRKGAEHAPWVEDWV<br>KGGEDRIFSLPSWGGWNKKTKKGLLLGGTAVAHLVQKKVITLKIFRSKVEKAGELLNNQQKMDELGIDVS<br>QARLMHAHMEGCLKEAEGLIKDSVTSAYVQQGSALDTAFSTYYWMWKADVTLANFGALNEMAFLYGQKPV<br>GQKKLLDTLKGTAYKWGSTLANMCATGDFNGERVHMHPGVFTPHRMSEMTAAIGAFPLSNPVKIKEGSAS<br>YRYLTNLKTSESNPAAKVVCELFNTHRRAHSDWNSPTSIVPPEHYFHQSLLERLGPFCHVSLVRGNALRV<br>NIVEEMGAGGTH
>BCU08953.1 nucleoprotein [Yezo virus]

MARLIEAKTREELDKWIHNLDTTHFLGLNTKYTKSLALQPCEPGSCPEAYISFDGKKSDELDAILSGALV
DSLKFAAPIPECAWNLTNKIFTEGMAWFNANKDQKCMAWDKKYDTLRSTLPSPEDVAQYQIAARKWRKDI
GYECSPNNGIMRGEVTKVYIAPKKYVNDMILMVTDMRSKRRHRLGITEEQEQEALSRKGAEHAPWVEDWV
KGGEDRIFSLPSWGGWNKKTKKGLLLGGTAVAHLVQKKVITLKIFRSKVEKAGELLNNQQKMDELGIDVS
QARLMHAHMEGCLKEAEGLIKDSVTSAYVQQGSALDTAFSTYYWMWKADVTLANFGALNEMAFLYGQKPV
GQKKLLDTLKGTAYKWGSTLANMCATGDFNGERVHMHPGVFTPHRMSEMTAAIGAFPLSNPVKIKEGSAS
YRYLTNLKTSESNPAAKVVCELFNTHRRAHSDWNSPTSIVPPEHYFHQSLLERLGPFCHVSLVRGNALRV
NIVEEMGAGGTH

>BCU08952.1 glycoprotein precursor [Yezo virus]

MLIMQCRDFLALWLLASVCLSESGDGSLNTTVKGEAPSPTTPAASAVAPTNKTLPEKTTTVTPGNTTRVA
TRPTSSPALSRWLAGLYSRNYEDFHRRVATGMVRSADGSARMESNILGYSGNLSKGEQLTTTLIRAVDPRIADVFQVK
RTVLAVKDRVAGYSATAAYNYLGKMVYNVDEDGVVTVTGLSDLGNSGYTLHLKTQGPHVPAD
YLRPGCEKLHNCCEGPEKQVDIFSLRSENIKVSSEPGSKYLSLAYLTGYLGFDARGCKAVVENHGSMYLL
TENSVEQVNTVNNGKVYPIIHIFIDRTDPGENCAISMCNLIPIPGATGGLPVRQTKKQLIKVSLGGPEHS
AFSRKLLSLRTQHNHAHQTPCNQPSHIMEIDQYKLHAPESSFAHSKSSICNGTLISNKDLSGVKGCYVVA
RATVMHNCKQADMEPFESRGKCLVDNKLVSCEASESCIRVKVNDTTFIKVKSNDYHNIIKCTKECLVPAP
KGSLVLVTCPDGEDIVLHTNKVDLDCPLLSYLGKPAIYVCRATYRPRVFYALIIWVVFGYSIGRIAFILT
FLLLWIFNRLTRTIIFRMDKQKDTCSGCGEFVDHTVKWARHDSCNSGSCPYCNKLSSLSKLKEHAKTCTS
KVKELEQDEKVLDIALVPKWSRRLESTIKAFSKTLSRIQWVVVVSCLLMYFLFHPVSGLSSNLKGKDFWDK
SITELEVCQPVCVYEEEYCTCPIQESPTVHNHSIHHIVKRSLLSLPVAGAPRDLDIEASWGTVHIENSYQ
PSVGGNHISLSWQSQNRVGSKVVVAGKSEAILRLDPRSGLKWSITSPDSSESRNIFVSILEYAQEYETQF
EYLTSNRKLGTWMHGVCTGTCPEKCGCKSHTCSSQKWENTRNWRCNPTWCWNIGGCTCCGADIDKPFQDW
ILTKWSTDYARTTVIACVEFEHGSRQCDLIDAGTTISLGGISVSFSGVTGITTMLPRQIALVHKAPGNHK
DFDLVKPIGMIDGTDLCKLQACTHGPAGDFQIYNLNGLVDNDVTNMNFWKTKTEAIKKDWMSWQGTVLQY
YCNPGDWPTCVMSGAVNDNSGAFENLWKRSAKLHQQYFFHSEKIDIRDGSPTLTLKGRPYMGGGQITALL
SVSGLELESKTVLPKGVKLHITMCEGCYGCATGITCSVSITLDSPDRYGLHLSSNTEGVIASETTLTCSA
GQVNDFKLRLFSSLPEDKICLRVREAPKESEDASSCLSVKLQPPEEVVLEHRSVLTSTGNSTCGKGFLSC
LGSNIAHFFGSIGTFLRDFFGSIWKGLVVIIPIVIILILLILFGPNLLSLIKVCISGRKSTIDRKVYYES
GKRELERYRQIIKEEEELLRRNDKKK

>BCU08951.1 RNA-dependent RNA-polymerase [Yezo virus]

MAGKLSKRPELTWEHRINNLFVASIVMPVDESFKVVRMPGDGKCFFYSVSHNMFGTIQGHTRVKWMVRDF
AEKHWDLLTEAKEFYRSREEYIRDLNRETYWGGSIEAEILSLRTNQPIIIWRAEDGYTITSASMWRGDSG
KGEINLIQSHNNHFDAAYPPELAGTTSGPAVSREDKTVLEAVGETIIDEDDESDTEMEVAKLEDTFLPTY
ESERIELLSKPTMPPVGPVRRLPARLEEMLVNEQTLPGKVGRHLNKLYSCNVSVLFINPGEALLIPSHSEVGGKVSISEL
GRLWLNPSKRIRKENLRTVIAISDQLMAYVDKGYVLRTFVPKTFISRHAGLVPPDLVRDM
TLTATVILLSTFLYKSRMVEKRTFIRACLRDEGRISGKKVSRILNKLTPHTLYNDPLSVVRQICLAMFGE
ECGRLVDLFSCLGPVSYLSLSSLELDSLTRNKYYQVLEELSRADTEDFDFSSKEISEIKATVDCCKALRE
ASDKESCEQATGTVLQILGISRQELPRRAKDNRREQEKLVINKFFNDRAISKFVSLQGRAYSGTNLSNIL
SYTHNLLMSKDKFKLTEDDVGQLELEQRKIRAMLAKEERVPIAIICSMMENRFNNLFKRLPPSCAQECEV
LFDSIRNAESYASAWRSALRLKGVAYEGMFALKYNCTYIPEDQKPTLSMIVQTYYPDKFVDFLDKTQKRP
EIRDYTPDFSICQMIVEHAEGLVPPKVSSVQLQAHDLEESGVKLRRTKEKAFPLPEVPIKELRSLDRLKE
LLLGTGKQNQPVHLKTEKYDESDCTVTSREIIAIEVGYQTDVDGKVFSDTHKWQEAIKLLSKLGIKALVH
ICADSSSTRLSDWWISPEDARLLKESISFLFHEMGENSPSDVTDIMVANISTTKVRAQLKSGSIVKTPVT
TKDVAAAWNAKLEKIRVRPTGAVLTDGLMDTVKKALVEGVTMDRASCEKVIEEVIIKNMDEIISLVEETK
NKHEVTRNYITANRLILGWLMEDIECCRCKACVAMVKDAIKGIASEQESVKYLCSQIQLSEHLPCCHTDL
PSVKPRSLFSTRCPDITTVRHMTGDGSTDDSELTELDKAIRLTLPGKTERERKIKRGLEQLIRLSMSDSH
IPCIKLPTGQILINRHAFNPTKVSEKRAAKNDEFRDSKLERLTKLLSRERLTGYSEKACKIMNSLLEKTH
LQGNSRCCLKPEWAQSVLMDLKTDRNEIDIATKLKETSEKRLNFIVNNDKIIPVSNSEIRQFIVDKSDTI
LNRKKNSKLFNLDCLIFKETVLEVLRRLNGTPYFDCVEIIATLTKLLLSFDWFQELVLYSKLCETFLQCC
TEFNRSGIKVRRVRHTSINLAIQLPSNKKENMKCCCYNLDFSRNKSTFLMNRRVAVLGAAYPYILIITTL
QCLQHQRCLDELTKEHASLQRDILTANAKLLDLLQSTLCKANLGSFEQAALEMRRHCKQTGNFLDHSSYD
MIVNTLAGLSVVFGLALGDTLLLNSQPFNKQIQNMRFTMLSGLSRLVSPLELGKKLSSSSRRIESGVSRL
YLQLISYCCVEQVEQNSENWKQFDLCPNVFMPSLSIFGMNTSGDRQLIFDIYLVHIYNKEMDNFDEGCIK
VLEETAEKHMSWELSLMNDLEKRTKKHERHARLLLGLPTLPRHELPTEDVIPEDSFSTSSSTSAISSTRS
FSSGKNIKTLFGRLKSEIKPFVVEDCLEVSRDELNDFKQVISDVGRGYSYEPNLQRLYKNVKTIIKENPN
HTFGSFELIQAFTELARKKFPSESIEKCRKDPRNWCSISEVVETTSIVSEPKHYISIKDALKTITGCETK
KFVKMLRNRLVKLGMEQGKDKIGEPSSFSLLESVDTLTEAQKENIRKGILEPSKLSFYNWRDIINKSLSDTLLTNDGN
MIYCWLKSMASGVKRGLKPYIKNLRYDREAKSDATDCLKLILSNEEVENLQSFIRVMKAIVD
PSRGEEEELYCVDLLYSVWVKFVSKVKFWKEIVESSLAAMTSCITDFENLIVMYTELNEQMKKYDCLSFT
REEIALKAQELRFLEQHGNNIVKFTNLIFFLCLACPWCMHYKSFELYLNKILETEPSRQLSPEAMKRLTS
LGPVNLIKSKWFVDLCSENGLSPPDAEITKHLDWLYRYCCALVSSNSEPLAATVNAMKPDEAVNTEQVLT

KLRGILAKYNLEDSDLDFKWTISMIANSNFEVAKKLTGRTTGERLPRSVRSKVIYEMIKLVGCSGMAVLQ
QLAFSTILNHNHEFFAVLAPKAQLGGHRDLLVQESGTKVVHATTEMFSRTLLSTTNDDGLTNSHLKEAIL
NEGLAKMMLSRRNHGRPVSEMDDDGCLQFFRVFCISGDNTKWGPIHCCSLFSGMMQQLLKDYPDWCNMYK
LTFLKNLYRKVEIPSAAIQKILTTFKTRLASKYKLETMRENELRKLLNESIEIWSDQPMIQFLIRTYLCK
GDMAMTCYNHMGQGIHHATSSILTSVMADGIELIIREFCKRTFPELTVEVAHAGSSDDYAKCITLSGAIS
KSTFERYERTFWPQMCRLKNLMAGVGRCCQMKDSAKTLCGDTMLEFYSEFMLSFRITPAVIKFIHTGLIN
SSVTSPQSMVQACQVSGQQAMYNSVPLITNICFTVLRQQMFFNHTEFFMREYGPLVHGLPSSFCRLYIPV
YSNLTGSSIAVEDSESISQDLKTLKDALPFLPNTKPLILSSELKDLVGSDDTKSTSSSHGSDKLTSGESS
EGSSSNFRFELKRELTETESEYLKSVHRDIDEASEMLVETRTKALFVGEEDYDGFPCFKRIKESNLIRSN
FEFVKLLEEKPILRTFGLVRSVICAIIAGYYRSFSSEGTEKTVKANLNRDENRVIEDPMIQLVPEKLRRE
LERLGLSRLSINELIGSGFSGLSLSEQIARRVVTMNCATEEYSSEIERLKQTLSSRNVIHGLAGGIKELS
LPIYTIFLKSYFFKDNVFFDHKDRWNTQHSTNYRDSTGKKLDGKIVTKFTVWLDRILSSTLSVSYKSQDF
EPSLFNSSLKLVDIIRMEEGPTDLSILSAELDVFLSEFRSLAMQFSEPNRLKLKIVESRPHTKELDANKV
IIVKSSIFNASEQVKIQNNPALIVGCMLSETIIMDVKPARVDYGGLMMDKHKVNCFYESIATVCNSITER
SKALEASDESPDLDEVSTSANTLTMLCRLIQKQNSRLVNFYMIKPMATDTEPTVTDLVSYGTKEGRLLLL
PEPNVEVSSFSVKYWKIMQSISAIANLNLTQQEKTDLLCSFLHWKPNKSIAMQDCPLMKHEMSMLSEFQG
SSLLATLSSEVMNIKNESTRHQLEDLIDFIKDPNLLVTKKPFIGTTAIFRTWGEGQKNGRFTFSSGNGES
TGIFIRGKLHIYLSLESEILLSEVEKQVLGWMNRRRTDIVVKEQHDYFLNLLPTYPMVPRKHTDGYIRGV
VINRNNPRMLGFTPAKYNSLVVRVKKRILTVKKSSKTCDCSEPRLYWTPNAITIIYDELTESATYHEEVVKLRQSVSMII
EPEKKVEVPSVAYQDFKVVIGKIRLDSEALLSSTVLLHYYLCHSPEEVRMELSSKSQRMI
ELLTGEGVIPGYRVSQKLYEDSRSIKKDLRVTPNLQLNIAGRLENYLNSTDARVSDWSEVQQILDESGCS
NIDVQLEQKTLEDRVSWRCLLSKISNRAPSMLTLRTLVSLVGSETIPNFLSGLLVHADVLDKLLRLGKSA
KNELSASSLDDRGLELLLSCTLYQLQVENKILHCQTYSTGSIFSLVTSKTFDSGNDMILGFTLDDEDFLS
FSITVPIPPLRKDASKREKAMAQGHALVAYNLFFDEVNNFQEVCSNTSLWRLQHFPDRSEIYMEFDYTKA
GNTTLLKLLVSSGLGMRDSILRLSPLIQFIATLVGRMLPEYLLEKAKEVKQSTNSCEFATEDDLLYLSEE
ELEPAKKEPEHGLFDLSF

>BCU08950.1 nucleoprotein [Yezo virus]

MARLIEAKTREELDKWIHNLDTTHFLGLNTKYTKSLALQPCEPGSCPEAYISFDGKKSDELDAILSGALV
DSLKFAAPIPECAWNLTNKIFTEGMAWFNANKDQKCMAWDKKYDTLRSTLPSPEDVAQYQIAARKWRKDI
GYECSPNNGIMRGEVTKVYIAPKKYVNDMILMVTDMRSKRRHRLGITEEQEQEALSRKGAEHAPWVEDWV
KGGEDRIFSLPSWGGWNKKTKKGLLLGGTAVAHLVQKKVITLKIFRSKVEKAGELLNNQQKMDELGIDVS
QARLMHAHMEGCLKEAEGLIKDSVTSAYVQQGSALDTAFSTYYWMWKADVTLANFGALNEMAFLYGQKPV
GQKKLLDTLKGTAYKWGSTLANMCATGDFNGERVHMHPGVFTPHRMSEMTAAIGAFPLSNPVKIKEGSAS
YRYLTNLKTSESNPAAKVVCELFNTHRRAHSDWNSPTSIVPPEHYFHQSLLERLGPFCHVSLVRGNALRV
NIVEEMGTGGNP

>BCU08949.1 glycoprotein precursor [Yezo virus]

MLIMQCRDFLALWLLASMCLSESGDGSLNTTVKGEAPSPTTPAASAVAPTNKTLPEKTTTVAPGNTTRVA
TRPTSSPALSRWLAGLYSRNYEDFHRRVATGMVRSADGSARMESNILGYSGNLSKGEQLTTTLIRAVDPR
IADVFQVKRTVLAVKDRVAGYSATAAYNYLGKMVYNVDEDGVVTVTGLSDLGNSGYTLHLKTQGPHVPAD
YLRPGCEKLHNCCEGPEKQVDIFSLRSENIKVSSEPGSKYLSLAYLTGYLGFDARGCKAVVENHGSMYLL
TENSVEQVNTVNSGKVYPIIHIFIDRTDPGENCAISMCNLIPIPGATGGLPVRQTKKQLIKVSLGGPEHS
AFSRKLLSLRTQHNHAHQTPCNQPSHIMEIDQYKLHAPESSFAHSKSSICNGTLISNKDLSGVKGCYVVA
RATVMHNCKQADMEPFESRGKCLVDNKLVSCEASESCIRVKVNDTTFIKVKSNDYHNIIKCTKECLVPAP
KGSLVLVTCPDGEDIVLHTNKVDLDCPLLSYLGKPAIYVCRATYRPRVFYALIIWVVFGYSIGRIAFILT
FLLLWIFNRLTRTIIFRMDKQKDTCSGCGEFVDHTVKWARHDSCNSGSCPYCNKLSSLSKLKEHAKTCTS
KVKELEQDEKVLDIALVPKWSRRLESTIKAFSKTLSRIQWVVVVSCLLMYFLFHPVSGLSSNLKGKDFWDK
SITELEVCQPVCVYEEEYCTCPIQESPTLHNHSIHHIVKRSLLSLPVAGAPRDLDIEASWGTVHIENSYQ
PSVGGNHISLSWQSQNRVGSKVVVAGKSEAILRLDPRSGLKWSITSPDSSESRNIFVSILEYAQEYETQF
EYLTSNRKLGTWMHGVCTGTCPEKCGCKSHTCSSQKWENTRNWRCNPTWCWNIGGCTCCGADIDKPFQDW
ILTKWSTDYARTTVIACVEFEHGSRQCDLIDAGTTISLGGISVSFSGVTGITTMLPRQIALVHKAPGNHK
DFDLVKPIGMIDGTDLCKLQACTHGPAGDFQIYNLNGLVDNDVTNMNFWKTKTEAIKKDWMSWQGTVLQY
YCNPGDWPTCVMSGAVNDNSGAFENLWKRSTKLHQQYFFHSEKIDIRDGSPTLTLKGRPYMGGGQITALL
SVSGLELESKTVLPKGVKLHITMCEGCYGCATGITCSVSITLDSPDRYGLHLSSNTEGVIASETTLTCSA
GQVNDFKLRLFSSLPEDKICLRVREAPKESEDASSCLSVKLQPPEEVVLEHRSVLTSTGNSTCGKGFLSC
LGSNIAHFFGSIGTFLKDFFGSIWKGLVVIIPIVIILILLILFGPNLLGLIKVCISGRKSTIDRKVYYES
GKRELERYRQIIKEEEELLRRNDKKK

>BCU08948.1 RNA-dependent RNA-polymerase [Yezo virus]

SSVTSPQSMVQACQVSGQQAMYNSVPLITNICFTVLRQQMFFNHTEFFMREYGPLVHGLPSSFCRLYIPV
YSNLTGSSIAIEDSESISQDLKTLKDALPFLPNTKPLILSSELKDLVGSDDTKSTSSSHGSDKLTSGESS
EGSSSNFRFELKRELTETESEYLKSVHRDIDEASEMLVETRTKALFVGEEDYDGFPCFKRIKESNLIRSN
FEFVKLLEEKPILRTFGLVRSVICAIIAGYYRSFSSEGTEKTVKANLNRDENRVIEDPMIQLVPEKLRRE
LERLGLSRLSINELIGSGFSGLSLSEQIARRVVTMNCATEEYSSEIERLKQTLSSRNVIHGLAGGIKELS
LPIYTIFLKSYFFKDNVFFDHKDRWNTQHSTNYRDSTGKKLDGKIVTKFTVWLDRILSSTLSVSYKSQDF
EPSLFNSSLKLVDIIRMEEGPTDLSILSAELDVFLSEFRSLAMQFSEPNRLKLKIVESRPHTKELDANKV
IIVKSSIFNASEQVKIQNNPALIVGCMLSETIIMDVKPARVDYGGLMMDKHKVNCFYESIATVCNSITER
SKALEASDESPDLDEVSTSANTLTMLCRLIQKQNSRLVNFYMIKPMATDTEPTVTDLVSYGTKEGRLLLL
PEPNVEVSSFSVKYWKIMQSISAIANLNLTQQEKTDLLCSFLHWKPNKSIAMQDCPLMKHEMSMLSEFQG
SSLLATLSSEVMNIKNESTRHQLEDLIDFIKDPNLLVTKKPFIGTTAIFRTWGEGQKNGRFTFSSGNGES
TGIFIRGKLHIYLSLESEILLSEVEKQVLGWMNRRRTDIVVKEQHDYFLNLLPTYPMVPRKHTDGYIRGV
VINRNNPRMLGFTPAKYNSLVVRVKKRILTVKKSSKTCDCSEPRLYWTPNAITIIYDELTESATYHEEVV
KLRQSVSMIIEPEKKVEVPSVAYQDFKVVIGKIRLDSEALLSSTVLLHYYLCHSPEEVRMELSSKSQRMI
ELLTGEGVIPGYRVSQKLYEDSRSIKKDLRVTPNLQLNIAGRLENYLNSTDARVSDWSEVQQILDESGCS
NIDVQLEQKTLEDRVSWRCLLSKISNRAPSMLTLRTLVSLVGSETIPNFLSGLLVHADVLDKLLRLGKSA
KNELSASSLDDRGLELLLSCTLYQLQVENKILHCQTYSTGSIFSLVTSKTFDSGNDMILGFTLDDEDFLS
FSITVPIPPLRKDASKREKAMAQGHALVAYNLFFDEVNNFQEVCSNTSLWRLQHFPDRSEIYMEFDYTKA
GNTTLLKLLVSSGLGMKDSILRLSPLIQFIATLVGRMLPEYLLEKAKEVKQSTNSCEFATEDDLLYLSEE
ELEPAKKEPEHGLFDLSF

>BCU08947.1 nucleoprotein [Yezo virus]
MARLIEAKTREELDKWIHNLDTTHFLGLNTKYTKSLALQPCEPGSCPEAYISFDGKKSDELDAILSGALV
DSLKFAAPIPECAWNLTNKIFTEGMAWFNANKDQKCMAWDKKYDTLRSTLPSPEDVAQYQIAARKWRKDI
GYECSPNNGIMRGEVTKVYIAPKKYVNDMILMVTDMRSKRRHRLGITEEQEQEALSRKGAEHAPWVEDWV
KGGEDRIFSLPSWGGWNKKTKKGLLLGGTAVAHLVQKKVITLKIFRSKVEKAGELLNNQQKMDELGIDVS
QARLMHAHMEGCLKEAEGLIKDSVTSAYVQQGSALDTAFSTYYWMWKADVTLANFGALNEMAFLYGQKPV
GQKKLLDTLKGTAYKWGSTLANMCATGDFNGERVHMHPGVFTPHRMSEMTAAIGAFPLSNPVKIKEGSAS
YRYLTNLKTSESNPAAKVVCELFNTHRRAHSDWNSPTSIVPPEHYFHQSLLERLGPFCHVSLVRGNALRV
NIVEEMGAGGTH

>BCU08946.1 glycoprotein precursor [Yezo virus]

MLIMQCRDFLALWLLASVCLSESGDGSLNTTVKGEVPSPTTPAASAVAPTNKTLPEKTTTVTPGNTTRVA
TRPTSSPALSRWLAGLYSRNYEDFHRRVATGMVRSADGSARMESNILGYSGNLSKGEQLTTTLIRAVDPR
IADVFQVKRTVLAVKDRVAGYSATAAYNYLGKMVYNVDEDGVVTVTGLSDLGNSGYTLHLKTQGPHVPAD
YLRPGCEKLHNCCEGPEKQVDIFSLRSENIKVSSEPGSKYLSLAYLTGYLGFDARGCKAVVENHGSMYLL
TENSVEQVNTVNNGKVYPIIHIFIDRTDPGENCAISMCNLIPIPGATGGLPVRQTKKQLIKVSLGGPEHS
AFSRKLLSLRTQHNHAHQTPCNQPSHIMEIDQYKLHAPESSFAHSKSSICNGTLISNKDLSGVKGCYVVA
RATVMHNCKQADMEPFESRGKCLVDNKLVSCEASESCIRVKVNDTTFIKVKSNDYHNIIKCTKECLVPAP
KGSLVLVTCPDGEDIVLHTNKVDLDCPLLSYLGKPAIYVCRATYRPRVFYALIIWVVFGYSIGRIAFILT
FLLLWIFNRLTRTIIFRMDKQKDTCSGCGEFVDHTVKWARHDSCNSGSCPYCNKLSSLSKLKEHAKTCTS
KVKELEQDEKVLDIALVPKWSRRLESTIKAFSKTLSRIQWVVVVSCLLMYFLFHPVSGLSSNLKGKDFWDK
SITELEVCQPVCVYEEEYCTCPIQESPTVHNHSIHHIVKRSLLSLPVAGAPRDLDIEASWGTVHIENSYQ
PSVGGNHISLSWQSQNRVGSKVVVAGKSEAILRLDPRSGLKWSITSPDSSESRNVFVSILEYAQEYETQF
EYLTSNRKLGTWMHGVCTGTCPEKCGCKSHTCSSQKWENTRNWRCNPTWCWNIGGCTCCGADIDKPFQDW
ILTKWSTDYARTTVIACVEFEHGSRQCDLIDAGTTISLGGISVSFSGVTGITTMLPRQIALVHKAPGNHK
DFDLVKPIGMIDGTDLCKLQACTHGPAGDFQIYNLNGLVDNDVTNMNFWKTKTEAIKKDWMSWQGTVLQY
YCNPGDWPTCVMSGAVNDNSGAFENLWKRSTKLHQQYFFHSEKIDIRDGSPTLTLKGRPYMGGGQITALL
SVSGLELESKTVLPKGVKLHITMCEGCYGCATGITCSVSITLDSPDRYGLHLSSNTEGVIASETTLTCSA
GQVNDFKLRLFSSLPEDKICLRVREAPKESEDASSCLSVKLQPPEEVVLEHRSVLTSTGNSTCGKGFLSC
LGSNIAHFFGSIGTFLKDFFGSIWKGLVVIIPIVIILILLILFGPNLLSLIKVCISGRKSTIDRKVYYES
GKRELERYRQIIKEEEELLRRNDKKK

>BCU08945.1 RNA-dependent RNA-polymerase [Yezo virus]

MAGKLSKRPELTWEHRINNLFVASIVMPVDESFKVVRMPGDGKCFFYSVSHNMFGTIQGHTRVKWMVRDF
AEKHWDLLTEAKEFYRSREEYIRDLNRETYWGGSIEAEILSLRTNQPIIIWRAEDGYTITSASMWRGDSG
KGEINLIQSHNNHFDAAYPPELAGTTSGPAVSREDKTVLEAVGETIIDEDDESDTEMEVAKLEDTFLPTY
ESERIELLSKPTMPPVGPVRRLPARLEEMLVNEQTLPGKVGRHLNKLYSCNVSVLFINPGEALLIPSHSE
VGGKVSISELGRLWLNPSKRIRKENLRTVIAISDQLMAYVDKGYVLRTFVPKTFISRHAGLVPPDLVRDM
TLTATVILLSTFLYKSRMVEKRTFIRACLRDEGRISGKKVSRILNKLTPHTLYNDPLSVVRQICLAMFGE
ECGRLVDLFSCLGPVSYLSLSSLELDSLTRNKYYQVLEELSRADTEDFDFSSKEISEIKATVDCCKALRE
ASDKESCEQATGTVLQILGISRQELPRRAKDNRREQEKLVINKFFNDRAISKFVSLQGRAYSGTNLSNIL
SYTHNLLMSKDKFKLTEDDVGQLELEQRKIRAMLAKEERVPIAIICSMMENRFNNLFKRLPPSCAQECEV
LFDSIRNAESYASAWRSALRLKGVAYEGMFALKYNCTYIPEDQKPTLSMIVQTYYPDKFVDFLDKTQKRP
EIRDYTPDFSICQMIVEHAEGLVPPKVSSVQLQAHDLEESGVKLRRTKEKAFPLPEVPIKELRSLDRLKE
LLLGTGKQNQPVHLKTEKYDESDCTVTSREIIAIEVGYQTDVDGKVFSDTHKWQEAIKLLSKLGIKALVH
ICADSSSTRLSDWWISPEDARLLKESISFLFHEMGENSPSDVTDIMVANISTTKVRAQLKSGSIVKTPVT
TKDVAAAWNAKLEKIRVRPTGAVLTDGLMDTVKKALVEGVTMDRASCEKVIEEVIIKNMDEIISLVEETK
NKHEVTRNYTTANRLILGWLMEDIECCRCKACVAMVKDAIKGIASEQESVKYLCSQIQLSEHLPCCHTDL
PSVKPRSLFSTRCPDITTVRHMTGDGSTDDSELTELDKAIRLTLPGKTERERKIKRGLEQLIRLSMSDSH
IPCIKLPTGQILINRHAFNPTKVSEKRAVKNDEFRDSKLERLTKLLSKERLTGYSEKACKIMNTLLEKTH
LQGNSRCCLKPEWAQSVLMDLKTDRNEIDIATKLKETSEKRLNFIVNNDKIIPVSNSEIRQFIVDKSDTI
LNRKKNSKLFNLDCLIFKETVLEVLRRLNGTPYFDCIEIIATLTKLLLSFDWFQELVLYSKLCETFLQCC
TEFNRSGIKVRRVRHTSINLAIQLPSNKKENMKCCCYNLDFSRNKSTFLMNRRVAVLGAAYPYILIITTL
QCLQHQRCLDELTKEHASLQRDILTANAKLLDLLQSTLCKANLGSFEQAALEMRRHCKQTGNFLDHSSYD
MIVNTLAGLSVVFGLALGDTLLLNSQPFNKQIQNMRFTMLSGLSRLVSPLELGKKLSSSSRRIESGVSRL
YLQLISYCCVEQVEQNSENWKQFDLCPNVFMPSLSIFGMNTSGDRQLIFDIYLVHIYNKEMDNFDEGCIK
VLEETAEKHMSWELSLMNDLEKRTKKHERHARLLLGLPTLPRHELPTEDVIPEDSFSTSSSTSAISSTRS
FSSGKNIKTLFGRLKSEIKPFVVEDCLEVSRDELNDFKQVISDVGRGYSYEPNLQRLYKNVKTIIKENPN
HTFGSFELIQAFTELARKKFPSESIEKCRKDPRNWCSISEVVETTSIVSEPKHYISIKDALKTITGCETK
KFVKMLRNRLVKLGMEQGKDKIGEPSSFSLLESVDTLTEAQKENIRKGILEPSKLSFYNWRDIINKSLSD
TLLTNDGNMIYCWLKSMASGVKRGLKPYIKNLRYDREAKSDATDCLKLILSNEEVENLQSFIRVMKAIVD
PSRGEEEELYCVDLLYSVWVKFASKVKFWKEIVESSLAAMTSCITDFENLIVMYTELNEQMKKYDCLSFT
REEIALKAQELRFLEQHGNNIVKFTNLIFFLCLACPWCMHYKSFELYLNKILETEPSRQLSPEAMKRLTS
LGPVNLIKSKWFVDLCSENGLSPPDAEITKHLDWLYRYCCALVSSNSEPLAATVNAMKPDEAVNTEQVLT
KLRGILAKYNLEDSDLDFKWTISMIANSNFEVAKKLTGRTTGERLPRSVRSKVIYEMIKLVGCSGMAVLQ
QLAFSTILNHNHEFFAVLAPKAQLGGHRDLLVQESGTKVVHATTEMFSRTLLSTTNDDGLTNSHLKEAIL
NEGLAKMMLSRRNHGRPVSEMDDDGCLQFFRVFCISGDNTKWGPIHCCSLFSGMMQQLLKDYPDWCNMYK
LTFLKNLYRKVEIPSAAIQKILTTFKTRLASKYKLETMRENELRKLLNESIEIWSDQPMIQFLIRTYLCKGDMAMTCYN
HMGQGIHHATSSILTSVMADGIELIIREFCKRTFPELTVEVAHAGSSDDYAKCITLSGAIS
