# Supplementary material for: Targeting Yezo Virus Structural Proteins for Multi-Epitope Vaccine Design Using Immunoinformatics Approach
Source: Viruses. 2024 Sep 3;16(9):1408. doi: 10.3390/v16091408 (PMC11437474; doi:10.3390/v16091408)
Supplement: Supplementary file 1 [file viruses-16-01408-s001.zip › Suppl. Table S2.pdf]

Supplementary table S2. Evaluating worldwide population coverage using MHC restriction data analysis.

Population/area	Class-I			Class-II			Class combined		
	coverage[a]	average_hit[b]	pc90[c]	coverage[a]	average_hit[b]	pc90[c]	coverage[a]	average_hit[b]	pc90[c]
Central Africa	90.66%	2.55	1.03	51.61%	1.04	0.21	95.48%	3.59	1.4
Central America	4.35%	0.09	0.1	44.62%	0.71	0.18	47.03%	0.79	0.19
East Africa	91.69%	2.89	1.09	55.11%	0.98	0.22	96.27%	3.87	1.55
East Asia	97.98%	3.51	1.69	56.03%	1.47	0.23	99.11%	4.98	2.31
Europe	98.94%	4.43	2.22	73.76%	2.11	0.38	99.72%	6.54	3.3
North Africa	96.43%	3.5	1.5	57.17%	1.3	0.23	98.47%	4.8	2.16
North America	98.61%	4.25	2.1	75.37%	2.35	0.41	99.66%	6.6	3.26
Northeast Asia	93.45%	2.95	1.25	39.85%	1.15	0.17	96.06%	4.1	1.65
Oceania	95.15%	2.75	1.26	45.62%	1.3	0.18	97.36%	4.06	1.62
South Africa	95.37%	2.72	1.3	5.91%	0.12	0.21	95.64%	2.84	1.34
South America	90.81%	2.57	1.03	46.1%	0.84	0.19	95.04%	3.41	1.31
South Asia	93.73%	2.86	1.24	64.26%	1.58	0.28	97.76%	4.45	2.0
Southeast Asia	92.66%	2.75	1.15	32.64%	0.88	0.15	95.05%	3.63	1.38
Southwest Asia	92.72%	3.16	1.17	34.64%	0.68	0.15	95.24%	3.84	1.44
West Africa	96.2%	3.34	1.44	45.61%	0.95	0.18	97.93%	4.29	1.93
West Indies	95.87%	3.18	1.33	60.48%	1.27	0.25	98.37%	4.45	2.01
World	97.79%	3.99	1.85	67.76%	1.95	0.31	99.29%	5.94	2.75
Average	89.55	3.03	1.34	50.38	1.22	0.23	94.32	4.25	1.86
Standard deviation	21.45	0.92	0.46	16.48	0.54	0.07	11.93	1.34	0.74

[a] Projected population coverage.

[b] Average number of epitope hits per HLA combination detected in the population.

[c] Minimum number of epitope matches required for recognition by 90% of the population for each HLA combination.
