# Supplementary material for: Targeting Yezo Virus Structural Proteins for Multi-Epitope Vaccine Design Using Immunoinformatics Approach
Source: Viruses. 2024 Sep 3;16(9):1408. doi: 10.3390/v16091408 (PMC11437474; doi:10.3390/v16091408)
Supplement: Supplementary file 1 [file viruses-16-01408-s001.zip › Suppl. Table S3.pdf]

Supplementary Table S3. Docking results of CTL and HTL epitopes with MHC I and II alleles.

Epitopes	Peptide	MHC-I	PDB ID	ΔG (kcal mol-1)	Kd (M) at ℃	Hydrogen bond
CTL epitopes	SIGRIAFIL	HLA-A*02:01	1B0G	-10.1	$4.2e^{-08}$	tyr7-ser275, tyr59-ser275, thr73-ala280, asp77-leu283, tyr99-gly277, trp147-le282, thr163-arg-278, thr163-arg278, tyr171-ser275
	LIIWVVFGY	HLA-A*29:02	2CLR	-10.6	$1.8e^{-08}$	tyr7-leu276, asn77-tyr284, arg114-phe282
	SIEAEILSL	HLA-A*02:01	2X4S	-9.0	$2.4e^{-07}$	tyr59-ser275, tyr59-ser275, glu63-leu276, gln70-val277, asp77-lys283, tyr99-val283, trp147-lys283, ala150-lys281, glu152-lys281, tyr159-ser275
	SLVEETKNK	HLA-A*03:01	3RL1	-8.5	$5.6e^{-07}$	tyr7-ser275, glu63-ile276, lys66-ile276, tyr159-ser275, tyr171-ser275, tyr171-ser275
	GTAYKWGST	HLA-B*15:01	2BVP	-10.8	$1.1e^{-08}$	glu76-ser284, asn80-thr285, asn80-ser284, lys146-ser284, lys146-thr285
	GLNTKYTKS	HLA-A*02:01	2X4S	-9.0	$2.4e^{-07}$	glu63-leu276, lys66-thr278, tyr116-thr281, trp147-lys282, tyr 159-gly275, tyr171-gly275
HTL epitopes	PVVFGYSIGRIAFIL	HLA-DRB1*13:02	1FV1	-7.2	$5.7e^{-06}$	Gln6-phe190, ser50-ile185, ser50-trp187, asn59-phe190, asp63-tyr192, asn66-ser193
	TFFYALIIWVVFGYS	HLA-DRB1*15:01	8TBP	-8.1	$1.1e^{-06}$	Asp63-tyr192, asn66-ser193, gln6-phe190, asn59-phe190, ser50-trp187, ice185-ser50, leu184-phe181
	DFGGSIEAEILSLRT	HLA-DRB1*04:01	5LAX	-8.4	$6.7e^{-07}$	Gln10-thr192, glu12-lys193, glu12-lys195, arg51-ile186, phe52-ile186, phe52-ser-187, ser54-ser187, ser54-val189, asp67-lys193,asn70-glu197,asn70-tyr199,arg77-glu197,
	ISLVEETKNKHEAYE	HLA-DRB1*12:01	3PDO	-5.8	$5.6e^{-05}$	Ser52-ile187, asn61-leu192, asn61-glu190
	KTTLKGTAYKWGSTL	HLA-DRB1*11:01	6CPL	-8.1	$1.1e^{-06}$	Glu1-gly180, gln6-his194,gln6-ser188,glu8-ala190,ser50-asn182, ser50-tyr185,ser50-asn182,glu52-lys187,glu52-lys184
	GLNTKYTKSLALQPH	HLA-DRB1*01:01	5V4N	-7.5	$2.3e^{-05}$	Gln8-ser193,gln8-thr194,asn61-thr194
