# Supplementary material for: Targeting Yezo Virus Structural Proteins for Multi-Epitope Vaccine Design Using Immunoinformatics Approach
Source: Viruses. 2024 Sep 3;16(9):1408. doi: 10.3390/v16091408 (PMC11437474; doi:10.3390/v16091408)
Supplement: Supplementary file 1 [file viruses-16-01408-s001.zip › Suppl. Table S4.pdf]

Supplementary table S4. The top five refine model of vaccine construct generated by GalaxyRefine.

Model	GDT-HA	RMSD	MolProbity	Clash score	Poor rotamers	Rama favored
Initial	1.0000	0.000	3.317	10.8	15.3	67.1
MODEL 1	0.9497	0.437	2.548	18.4	1.7	87.7
MODEL 2	0.9459	0.431	2.677	18.9	2.3	86.7
MODEL 3	0.9400	0.449	2.409	18.4	1.2	88.0
MODEL 4	0.9433	0.445	2.621	18.0	2.0	86.2
MODEL 5	0.9427	0.437	2.552	18.4	1.7	87.5
